# Evolutionary transcriptomics reveals the origins of olives and the genomic changes associated with their domestication

**DOI:** 10.1111/tpj.14435

**Published:** 2019-07-11

**Authors:** Muriel Gros‐Balthazard, Guillaume Besnard, Gautier Sarah, Yan Holtz, Julie Leclercq, Sylvain Santoni, Daniel Wegmann, Sylvain Glémin, Bouchaib Khadari

**Affiliations:** ^1^ AGAP, University Montpellier, CIRAD, INRA Montpellier SupAgro Montpellier France; ^2^ Laboratoire EDB UMR5174 CNRS‐UPS‐IRD Toulouse France; ^3^ Department of Biology University of Fribourg Fribourg Switzerland; ^4^ Swiss Institute of Bioinformatics Fribourg Switzerland; ^5^ CNRS Université de Rennes ECOBIO (Ecosystèmes, biodiversité, évolution) − UMR 6553 F‐35000 Rennes France; ^6^ Department of Ecology and Genetics Evolutionary Biology Centre Uppsala University Uppsala Sweden; ^7^ Conservatoire Botanique National Méditerranéen UMR AGAP Montpellier France; ^8^ Present address: New York University Abu Dhabi (NYUAD), Center for Genomics and Systems Biology Saadiyat Island Abu Dhabi United Arab Emirates

**Keywords:** artificial selection, differential expression analysis, domestication, *Olea europaea* (olive tree), perennial crop, RNA‐sequencing, transcriptomics

## Abstract

The olive (*Olea europaea* L. subsp. *europaea*) is one of the oldest and most socio‐economically important cultivated perennial crop in the Mediterranean region. Yet, its origins are still under debate and the genetic bases of the phenotypic changes associated with its domestication are unknown. We generated RNA‐sequencing data for 68 wild and cultivated olive trees to study the genetic diversity and structure both at the transcription and sequence levels. To localize putative genes or expression pathways targeted by artificial selection during domestication, we employed a two‐step approach in which we identified differentially expressed genes and screened the transcriptome for signatures of selection. Our analyses support a major domestication event in the eastern part of the Mediterranean basin followed by dispersion towards the West and subsequent admixture with western wild olives. While we found large changes in gene expression when comparing cultivated and wild olives, we found no major signature of selection on coding variants and weak signals primarily affected transcription factors. Our results indicated that the domestication of olives resulted in only moderate genomic consequences and that the domestication syndrome is mainly related to changes in gene expression, consistent with its evolutionary history and life history traits.

## Significance Statement

We revisited the evolutionary history of the iconic olive tree by reconstructing a model of domestication and inferring the genomic changes associated with its cultivation. Although more challenging than studying annual crops, further studies of the genomic of the domestication process in perennial should broaden our understanding of the genomic bases and dynamics of adaptation.

## Introduction

The olive tree (*Olea europaea* L. subsp. e*uropaea* var. *europaea*) constitutes a cornerstone of Mediterranean culture by its multiple past and present uses and its omnipresence in traditional agrosystems (Loumou and Giourga, 2003). Historically, olives were restricted to the Mediterranean basin, but current cultivation includes also the Americas and Australia. By 2017, more than 10 million hectares were devoted to olive cultivation globally, with more than 90% in the Mediterranean area (FAO, 2018). Hundreds of clonally propagated cultivars have been described (Bartolini *et al*., 1998), but only a few are cultivated on a large scale, such as ‘Leccino’ or ‘Picual’. Today, the olive tree is one of the most important oil‐producing plant species, and demand for its oil is still increasing due to its nutritional quality (Kalua *et al*., 2007). Over 20 million tonnes of olives were harvested in 2017, and the production is in constant growth (FAO, 2018).

The domestication history of olives, however, remains unresolved and its origins are partly lost in the mists of time (Besnard *et al*., 2018). Olive domestication is a still ongoing process involving the selection and vegetative propagation of individuals of high agronomical value, but also the establishment of orchards with various cultivation practices. While archaeological remains indicate that Mediterranean wild olives (also called oleasters; var. *sylvestris*) were already used during the Palaeolithic (Kislev *et al*., 1992), cultivation and early domestication efforts are supposed to have started only about 8000–6000 years bp in the Levant, where the olive oil trade has been developed during the Bronze Age (Liphschitz *et al*., 1991; Galili *et al*., 1997; Terral *et al*., 2004; Kaniewski *et al*., 2012).

Botanical and genetic data attest that cultivated olives mostly derive from oleasters (Besnard *et al*., 2001b, 2013a, 2013b; Terral *et al*., 2004; Breton *et al*., 2006; Carrión *et al*., 2010; Díez *et al*., 2015). Oleasters are found all over the Mediterranean basin and are partitioned in two main gene pools in the East and the West, according to plastid and nuclear markers (Besnard *et al*., 2001b, 2013b; Breton *et al*., 2006; Díez *et al*., 2015). Likewise, the cultivated germplasm shows some degrees of geographic differentiation between the East, Central and West of the Mediterranean basin; yet, extensive admixture between these gene pools has occurred, especially in the western part of its distribution (Besnard *et al*., 2001a, 2013a; Breton *et al*., 2009, 2006; Díez *et al*., 2015; Lumaret *et al*., 2004). Whether these two gene pools were independently domesticated remains highly debated (Besnard *et al*., 2018). Some studies on a few dozens of genetic markers support a main domestication event in the East, followed by diffusion to the West where this genepool was introgressed by local oleaster populations (Besnard *et al*., 2013a,b; Khadari and El Bakkali, 2018). Other studies sustain at least two independent domestications in the East and Central Mediterranean basin (Breton *et al*., 2006; Díez *et al*., 2015).

Even less is known about the genetic bases underlying the domestication of olives. In many crops, domestication genes were recently identified through QTL mapping, genome‐wide association studies, candidate gene approaches and more recently population genomic screens for signatures of selection (Doebley *et al*., 2006; Meyer and Purugganan, 2013; Gepts, 2014). Despite examples of important protein‐altering mutations (e.g. Olsen and Purugganan, 2002; Olsen and Wendel, 2013), these studies highlight the predominant role of regulatory mutations affecting either coding (i.e. transcription factors) or non‐coding regions (i.e. *cis*‐ regulatory elements) (Meyer and Purugganan, 2013; Lemmon *et al*., 2014; Martínez‐Ainsworth and Tenaillon, 2016; Swinnen *et al*., 2016). For instance, the overexpression of the *tb1* gene in maize is responsible for its apically dominant architecture (Doebley *et al*., 1995; Studer *et al*., 2011), and the rewiring of the expression of several genes in oilseed rape (*Brassica napus*), increased seed oil content (Liu *et al*., 2015). However, most of these findings stem from annuals, while the genetic architecture of perennial domestication remains elusive (Gaut *et al*., 2015). In olives, QTL analyses have identified molecular markers associated with fruit yield and flowering variables (Ben Sadok *et al*., 2013; Atienza *et al*., 2014), but the genetic basis of other phenotypic changes associated with domestication attested by the archaeobotanical record, such as an increase in fruit size or oil content (Galili *et al*., 1997), is still unknown.

Here we present a study based on transcriptome sequences of wild and cultivated olive trees that aims at clarifying the evolutionary history of olives and identifying the molecular changes associated with its domestication. Transcriptomic data at the population level are powerful to study both the changes in regulation, through the study of gene expression differential, as well as the change in protein‐coding genes, with no or minor biases when compared with genome‐wide data (Ray *et al*., 2011; Romiguier *et al*., 2014). It has previously been used successfully to study the domestication process in several crops, including African rice (Nabholz *et al*., 2014), common bean (Bellucci *et al*., 2014) and tomato (Koenig *et al*., 2013; Sauvage *et al*., 2017).

To date, genome assemblies of olives and oleasters are available (Barghini *et al*., 2014; Cruz *et al*., 2016; Unver *et al*., 2017), along with several transcriptome assemblies from different tissues of olives (Muñoz‐Mérida *et al*., 2013; Parra *et al*., 2013; Carmona *et al*., 2015; Iaria *et al*., 2016; Sarah *et al*., 2017). While transcriptomic studies have highlighted differences in gene expression in response to cold acclimation (de la O Leyva‐Pérez *et al*., 2015) or salt stress (Mousavi *et al*., 2019), neither the domestication history of olives nor the genetic architecture of its domestication syndrome have been studied with such data so far. We therefore generated transcriptomes of 39 cultivated accessions and 27 oleasters, which we used to refine the scenario of domestication and expansion. We further inferred the genomic changes associated with olive domestication using a two‐step strategy: we first contrasted transcriptome‐wide expression levels in wild and cultivated olives to identify changes in gene expression. Second, we scanned the transcriptomic sequences for signatures of selection to identify alleles potentially associated with domestication. The identification of regions under positive selection is methodologically challenging as selection and demography leave very similar signatures in the genome (Stephan, 2016). This is particularly problematic for perennial crops that have complex domestication history with possibly more than one origin and ongoing wild‐cultivated gene flows (Miller and Gross, 2011). We therefore made use of the demographic model inferred here to properly look for signatures of selection in the olive transcriptome.

## Results

### RNA‐sequencing and variant detection

RNA‐seq data were generated for 66 Mediterranean olive accessions and two accessions of *O. e. cuspidata* using an Illumina HiSeq2000 sequencer, resulting in a total of 2 billion reads of 100 bp with an average of 30 million reads per individual (Table S1). After removing low‐quality reads, trimming low‐quality bases and keeping only reads mapping to the reference transcriptome of var. ‘Arbequina’ (Sarah *et al*., 2017) with high quality (Methods S2), the average and median depth were 33.4× and 13.1× per accessions, respectively (Table S1). Variant calling led to 583 455 single nucleotide polymorphisms (SNPs) when the outgroup *O. e. cuspidata* was included, and to 536 341 SNPs when it was excluded. We eliminated four cultivated olive accessions identified as clones based on inferred relatedness (Figure S1) and therefore kept a total of 62 unique olive genotypes for downstream analyses. Variant calling on those samples resulted in 536 113 SNPs over 25 835 contigs.

### Evolutionary history of olive tree domestication

#### Structure of nuclear and chloroplastic diversity

**Figure 1:**
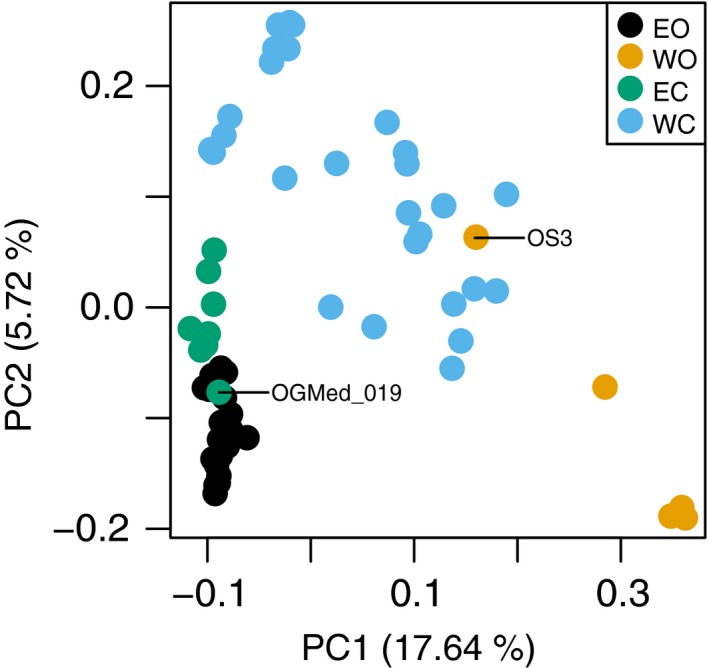
Principal component analysis plot (PCA) of the first two factors based on 536 341 SNPs genotyped in 62 olive accessions. Accessions are coloured according to the population they belong to as defined using their geographic origin, these results and the results from the admixture analysis and chloroplast genotyping (see text). EO, eastern oleasters; WO, western oleasters; EC, eastern cultivated olives; WC, western cultivated olives.

**Figure 2:**
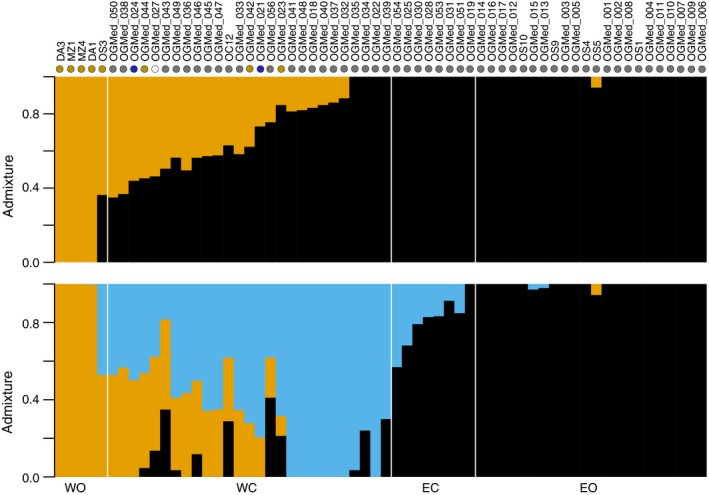
Admixture proportion in wild and cultivated olives (*n* = 62) with *K *=* *2 and 3 inferred from 536 341 SNPs. On the top of the graph, coloured dots indicate the chlorotype of each accession. Grey: E1; brown: E2; blue: E3; white: L1 (Data S1). Accessions were split in four different populations: WC and EC for western and eastern oleasters, respectively, based on their geographic origin and WC and EC for western and eastern cultivars, respectively, based on PCA (Figure 1) and structure results (this figure) along with chloroplastic genotyping data (Data S1).

We examined population structure in our olive transcriptomic data using principal component analysis (PCAs) (Figure 1) and NGSadmix (Figure 2). A strong geographic structure between the West and East of the Mediterranean Basin was observed along with a subtler differentiation between wild and cultivated accessions (Figures 1, 2 and S2–S6). Eastern and western oleasters clustered into distinct groups in the admixture analysis (Figure 2; Appendix S1), and stretched the first principal component of the PCA (Figure 1). Many cultivars sampled in the East of the Mediterranean Basin could hardly be differentiated from eastern oleasters in the admixture analysis (Figure 2). They appeared distinct although close in the PCA with only one cultivated accession falling among the eastern oleasters (OGMed_019: cultivar Zard from Iran; Figure 1). The oleaster OS3 from Morocco clustered with cultivated accessions (Figure 1) and displayed a mixed ancestry (Figure 2). The distinction between western and eastern accessions was also supported by chloroplast lineages (Figure S7). Eastern and western oleasters displayed chlorotypes from lineages E1 and E2, respectively. Most cultivars carried a chlorotype from lineage E1, but some western cultivars carried chlorotypes from lineages E2 and E3, which had been previously reported to be western (Besnard *et al*., 2013b).

Based on these results, we split cultivated accessions into two populations: (1) a western cultivated population comprised of 27 accessions with <50% of ancestry from the eastern oleaster cluster in the admixture analysis with *K *=* *3, and either chlorotypes from lineages E1, E2, E3 or L1 (Figure 2); and (2) an eastern cultivated population comprised of the remaining eight accessions that displayed ancestry mainly from the eastern oleaster clade (>50% with *K *=* *3) and the eastern chlorotype lineage E1. This assignment matches the presumed geographic origin of cultivars (Table S1), with the eastern population including all cultivars from Iran, Syria, Lebanon, Egypt and Cyprus, and the western population all cultivars from Greece and further to the West. The only exception was accession OGMed_032 (cultivar Stanbouli), which was assigned to the western population while its presumed origin is Syria. But this misclassification was previously noted in an a microsatellite analysis (Haouane *et al*., 2011).

#### Genetic diversity

When grouped together, wild accessions displayed on average more diversity than cultivated accessions (Table 1), as predicted for crops (Gepts, 2004). We observed a loss of diversity of about 14% and a slight increase in Tajima's *D* in eastern cultivated accessions compared to eastern oleasters, compatible with a weak to moderate bottleneck during domestication. Western cultivated accessions displayed more variability than eastern cultivated accessions in all diversity estimators as well as a more positive Tajima's *D* than the other populations, likely due to its admixed origin (Figures 1 and 2).

**Table 1:** Summary of population diversity statistics calculated on cultivated (1) and wild accessions (2) and on western and eastern subpopulations (a and b, respectively)

Population	No. accessions	Site class	% π	% θ_*W*_	Tajima's *D*	*H*_*E*_	*H*_*O*_	*C*
(1) Cultivated olives	35	Synonymous	0.969	0.807	0.243	0.0110	0.00792	0.614
		Non‐synonymous	0.374	0.339	0.160	0.0041	0.00263	
		All sites	0.576	0.490	0.423	0.00612	0.00395	
a) Western cultivated	27	Synonymous	0.987	0.772	0.408	0.0121	0.00792	0.615
		Non‐synonymous	0.380	0.317	0.411	0.00425	0.00263	
		All sites	0.582	0.463	0.693	0.00657	0.00395	
b) Eastern cultivated	8	Synonymous	0.789	0.681	0.215	0.00920	0.00792	0.624
		Non‐synonymous	0.297	0.268	0.224	0.00363	0.00263	
		All sites	0.448	0.392	0.434	0.00511	0.00395	
(2) Oleasters	27	Synonymous	1.001	1.014	−0.209	0.0114	0.00834	0.615
		Non‐synonymous	0.385	0.448	−0.494	0.00481	0.00263	
		All sites	0.589	0.621	−0.270	0.00636	0.00398	
a) Western oleasters	5	Synonymous	0.904	0.843	0.059	0.00936	0.00822	0.629
		Non‐synonymous	0.335	0.322	0.017	0.00390	0.00263	
		All sites	0.497	0.468	0.197	0.00496	0.00394	
b) Eastern oleasters	22	Synonymous	0.866	0.842	−0.143	0.0106	0.00837	0.617
		Non‐synonymous	0.332	0.368	−0.393	0.00371	0.00263	
		All sites	0.508	0.512	−0.178	0.00577	0.00398	

π, Mean number of pairwise difference; θ_*W*_, number of segregating sites; *H*_*E*_, expected heterozygosity; *H*_*O*_, observed heterozygosity; *C*, selective constraint.

The fixation index (*F*_*ST*_; Table 2) was the highest between western oleasters and eastern cultivated olives (21.8%). While the two oleaster populations were highly differentiated (16.7%), the two cultivated populations were much more similar (5.77%). Eastern cultivated olives appeared to be genetically very close to eastern oleasters (1.31%).

**Table 2:** Pairwise genetic differentiation (*F*_*ST*_) and standard deviation between pairs of populations

	C	WO	WC	EC	EO
O	0.0243 ± 0.0559	/	0.0319 ± 0.0657	0.0107 ± 0.0838	/
WO	0.118 ± 0.239	0.000	0.116 ± 0.220	0.218 ± 0.307	0.167 ± 0.313
WC	/	0.116 ± 0.220	0.000	0.0559 ± 0.115	0.0577 ± 0.0883
EC	/	0.218 ± 0.307	0.0559 ± 0.115	0.000	0.0131 ± 0.0862
EO	0.0434 ± 0.0729	0.167 ± 0.313	0.0577 ± 0.0883	0.0131 ± 0.0862	0.000

C, cultivars (*n* = 35); EO, eastern oleasters (*n* = 22); EW, eastern cultivated olives (*n* = 8); O, oleasters (*n* = 27); WC, western cultivated olives (*n* = 27); WO, western oleasters (*n* = 5).

#### Inference of olive population splits and mixtures

**Figure 3:**
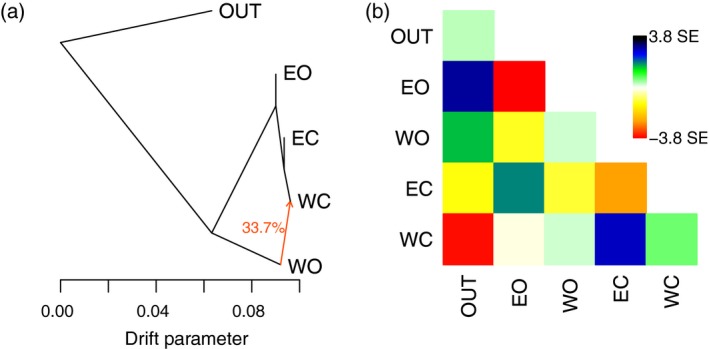
Patterns of divergence and migration between four olive populations with one migration event, as inferred by TreeMix. (a) Relationships between four populations; (b) Corresponding residuals of the model. OUT,* O. e. cuspidata* accessions; WO, western oleasters; EC, eastern cultivated accessions; WC, western cultivated accessions; EO, eastern oleasters.

We identified patterns of divergence and migration between the four olive populations with TreeMix, using the *O. e. cuspidata* individuals to root the tree (Figure 3). One migration event was required to explain >99.9% of the variance in the data (Figures S8 and S9) and results were consistent across five separate runs. The resulting tree confirms an initial domestication event in the East as eastern and western cultivars clustered together, separated from eastern oleasters by a very short branch only. However, the analysis also confirmed the admixed origin of western cultivars, which were estimated to have received 33.73% of their gene pool from western oleasters (Figure 3). To test for the robustness of this admixture signal, we repeated the analysis without the introgressed western oleaster (OS3), resulting in a 28.77% contribution of western oleasters to western cultivars. We further repeated the analysis by separating pure from admixed western cultivars based on the admixture analysis with *K *=* *3. Both western cultivated populations clustered with the eastern oleasters and cultivars and we inferred a 40.60% contribution of western oleasters to the western cultivars (Figures S10 and S11).

### Gene expression profiling

To avoid issues related to the independent construction of libraries (Conesa *et al*., 2016), we focused on the first sequencing batch, which contained data from all four populations studied here (Table S1). These data were generated by RNA‐sequencing both inflorescences and leaves.

#### Population structure and diversity in gene expression

**Figure 4:**
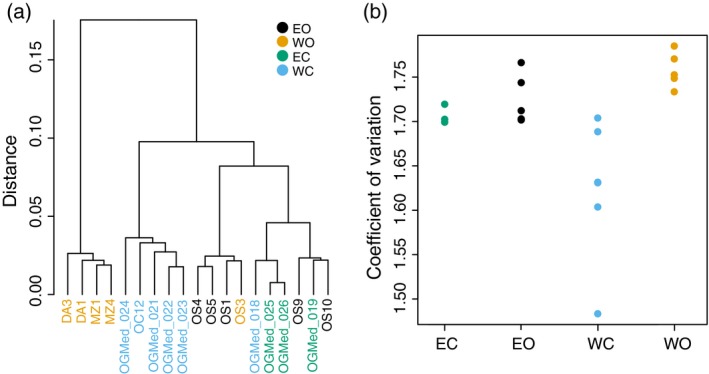
Gene expression data of 19 wild and cultivated olives over 40 831 transcripts. (a) Dendrogram of hierarchical clustering from gene expression data; (b) Gene expression diversity calculated as the level of gene expression variation for each sample. EO, eastern oleasters; WO, western oleasters; EC, eastern cultivars; WC, western cultivars.

We obtained expression profiles for each of the 19 accessions (Table S1) after filtering transcripts based on high intrapopulation variance and low read numbers, leading to 40 831 transcripts (90%). Hierarchical clustering on the expression profiles clustered the two known clones (OGMed_025 and OGMed_026) together, confirming the reliability of our expression analysis. We identified three major groups (Figure 4a). The first and most divergent group consisted of the western oleasters. The second group consisted of the eastern oleasters and eastern cultivars, which could not be separated into individual groups. The third groups consisted of western cultivars, which appeared intermediate between the other groups, in line with their admixed origin (Figures 1, 2, 3). Two samples, however, did not match this general pattern as the expression profile of both the western cultivar OGMed_018 and the introgressed western oleaster OS3 appeared nested within the eastern group. Although we observed a lower gene expression diversity in cultivars compared to oleasters, this difference was not significant (Student's *t*‐test, *P *=* *0.056 and 0.093 for eastern and western accessions, respectively; Figure 4b).

#### Transcriptional differences in olives

**Figure 5:**
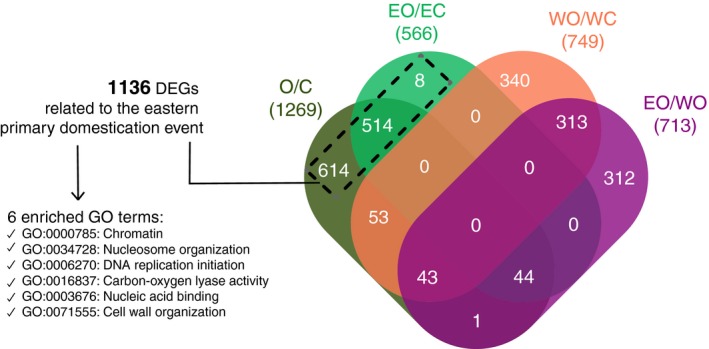
Overlap of differentially expressed genes (DEGs) identified in four differential expression analyses including a total of 19 wild and cultivated accessions. O/C: comparison between all oleasters and all cultivated accessions; EO/EC: comparison between eastern oleasters and eastern cultivars; WO/WC: comparison between western oleasters and western cultivars; EO/WO: comparison between eastern oleasters and western oleasters. Numbers in parentheses indicate the total number of DEGs for each of these four analyses. We identified 1136 genes differentially expressed in relation to the domestication process. These were DEGs either when comparing all oleasters and all cultivars or when comparing eastern oleasters and eastern cultivars or both but were removed from this list DEGs related to eastern/western geographic differentiation as identified by the analysis EO/WO and DEGs in relation to the divergence between western oleasters and western cultivars (WO/WC).

Differentially expressed genes (DEGs) were identified in six pairwise comparisons (Table 3). Interestingly, the divergence in gene expression between western and eastern accessions (1280 DEGs) is comparable with that between oleasters and cultivated accessions (1269 DEGs). Additionally, the lowest number of DEGs among these six comparisons was found when comparing eastern oleasters to eastern cultivars, supporting a low differentiation between these two populations. Most of the DEGs identified between eastern oleasters and eastern cultivars were also divergent when comparing all wild and all cultivated olives (Figure 5). But only a fraction of the DEGs between western oleasters and western cultivated accessions were also divergent between all wild and all cultivated accessions.

**Table 3:** Summary of the transcriptome‐wide differential of expression analysis performed on 40 831 transcripts profiled in 19 wild and cultivated olives

Pairwise comparison	No. DEGs (total, upregulated, downregulated)	Enriched GO
Oleasters versus cultivated olives	1269	GO:0000785: Chromatin; GO:0034728: Nucleosome organization; GO:0016837: Carbon‐oxygen lyase activity, acting on polysaccharides; GO:0006270: DNA replication initiation
	543	/
	726	GO:0000785: Chromatin; GO:0034728: Nucleosome organization; GO:0006270: DNA replication initiation; GO:0016837: Carbon‐oxygen lyase activity, acting on polysaccharides; GO:0006563: l‐serine metabolic process
Eastern oleasters versus eastern cultivated accessions	566	/
	233	/
	333	GO:0006270: DNA replication initiation; GO:0016837: Carbon‐oxygen lyase activity, acting on polysaccharides
Western oleasters versus western cultivated accessions	749	/
	257	/
	492	/
Western accessions versus eastern accessions (both wild and cultivated)	1280	GO:0009648: Photoperiodism; GO:0009791: Post‐embryonic development; GO:0004486: Methylenetetrahydrofolate dehydrogenase activity; GO:0019238: Cyclohydrolase activity;
	433	GO:0010557: Positive regulation of macromolecule biosynthetic process
	847	GO:0009648: Photoperiodism; GO:0004486: Methylenetetrahydrofolate dehydrogenase activity; GO:0019238: Cyclohydrolase activity
Eastern oleasters versus western oleasters	713	GO:0004486: Methylenetetrahydrofolate dehydrogenase activity; GO:0019238: Cyclohydrolase activity
	242	/
	471	GO:0004486: Methylenetetrahydrofolate dehydrogenase activity; GO:0019238: Cyclohydrolase activity
Eastern cultivated accessions versus western cultivated accessions	571	/
	195	/
	376	/

We identified primary domestication DEGs as genes differentially expressed between all wild and all cultivated accessions and/or eastern oleasters and eastern cultivated accessions, excluding genes differentially expressed when comparing eastern with western accessions (oleasters or cultivars) to correct for the high geographic differentiation in olives. Among these 1136 primary domestication DEGs, six Gene Ontology (GO) terms were significantly enriched (*P *<* *0.05), of which four were related to transcriptional activity (Figure 5).

### Detection of loci under selection in cultivated olives

No sign of relaxation of the selective constraint (*C*) on non‐synonymous sites was detected in domesticated olives with all four olive populations showing very similar values (Table 1).

To focus on the primary domestication event that took place in the East, and dispose of the effect of gene flows between western oleasters and the cultivated olive accessions, we first scanned the transcriptome for signatures of selection using eastern accessions only. Neither BayeScan nor PCAdapt detected outlier SNPs when comparing eastern oleasters with eastern cultivated accessions (Table 4 and Figure S12), nor did BayeScan identify outliers when including western cultivated accessions in the cultivated population.

**Table 4:** Selection scans performed on the RNA‐seq olive dataset with BayeScan and PCAdapt

Software	Population pairwise comparisons	Parameter	Threshold	No. outlier SNPs	No. contigs with outliers	No. enriched GO terms
BayeScan	Oleasters vs. cultivated	100	FDR = 0.05	30	22	0
		1000	FDR = 0.05	7	7	0
	Eastern oleasters vs. all cultivated	100	FDR = 0.05	0	/	/
		1000	FDR = 0.05	0	/	/
	Eastern oleasters vs. eastern cultivated	100	FDR = 0.05	0	/	/
		1000	FDR = 0.05	0	/	/
	Eastern accession vs. western accessions	100	FDR = 0.05	1	1	0
		1000	FDR = 0.05	0	/	/
	Eastern cultivars vs. western cultivars	100	FDR = 0.05	0	/	/
		1000	FDR = 0.05	0	/	/
	Eastern oleasters vs. western oleasters	100	FDR = 0.05	0	/	/
		1000	FDR = 0.05	0	/	/
PCAdapt	All olives	factors 1‐2	FDR = 0.01	3211	1119	298
	All olives	factor 2	top 1%	3000	1649	97
	Eastern olives	factor 1	FDR = 0.01	0	/	/

The parameter column in the case of BayeScan indicates the value set for the prior odds. For PCAdapt, it indicates the principal component used to find outlier loci.

Under a model contrasting 27 wild against 35 cultivated olives, BayeScan identified 30 and seven SNPs as potentially under selection with a prior odds of 100 and 1000, respectively (Table 4 and Table S2; Figure S13). Nevertheless, these outliers could reflect the high differentiation between western cultivars and eastern oleasters that is not due to domestication but to their admixture with the highly differentiated western oleaster gene pool. To correct the candidate genes list, we therefore identified SNPs and contigs exhibiting large *F*_*ST*_ between eastern and western accessions. Comparing all eastern with all western accessions, BayeScan identified a single outlier SNP, while when taking cultivars or oleasters only, the comparisons led to no outlier SNP. This single outlier did not overlap with the 30 and seven from the oleaster/cultivar comparison indicating that the oleaster/cultivar comparison could actually provide insights into the domestication event. Nevertheless, no enriched GO term was identified among these outliers SNPs (Table 4).

PCAdapt identified 3211 candidate SNPs that were atypically associated with both PC1 and PC2 (FDR < 0.01) when ran on all accessions together. PC1 captures the geographic partitioning of olive diversity while PC2 actually relates to the distinction between oleasters and cultivated olives (Figure 1). Therefore, to identify SNPs related to domestication, but not to the geographic differentiation, we focused on the top 1% hits associated to PC2 only. These 3000 SNPs were distributed over 1648 transcripts enriched for 97 GO terms (Figure S14). Enriched GO terms for cellular components were mostly related to chromosome organization, transcription complex as well as endomembrane structures (lipid bilayer) while enriched GO terms for molecular function were mostly related to protein synthesis (mRNA transcription and translation) and growth (growth factor binding, tubulin binding and cytoskeletal protein binding).

**Figure 6:**
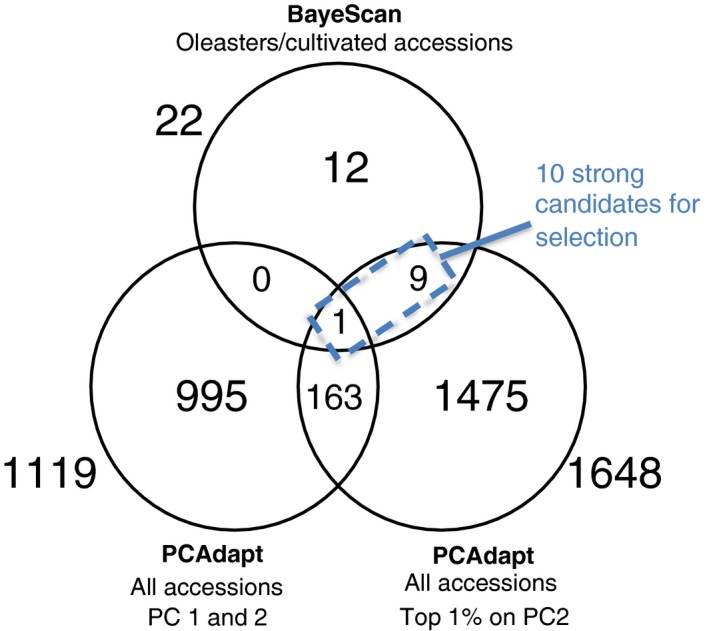
Overlap between selection scan analyses. The 10 contigs found as outliers in both BayeScan (all accessions) and PCAdapt (all accessions, top 1% on PC2) can be considered as strong candidates for selection.

Ten of these outlier transcripts also contained an SNP identified by BayeScan and are therefore the strongest candidates for selection (Figure 6). Their sequence similarity to the nucleotide collection of NCBI was identified with *blastn* using default parameters (Zhang *et al*., 2000; Morgulis *et al*., 2008): three transcripts are proteins involved in transcriptional and translational activities and two are involved in cell cycle (Table S3).

### Differentially expressed transcripts do not show more sign of selection

We investigated whether genes showing divergent expression in relation to the primary eastern domestication event were also identified as likely targets of selection. The intersection of the top 1% transcripts associated to PC2 in the PCAdapt analysis combining all samples (3000 SNPs on 1648 contigs) with the DEGs in all, eastern only and western only accessions (Table 3) led to, respectively, 73, 35 and 32 overlapped contigs. These numbers are, however, not different from what is expected by chance (hypergeometric test; *P *=* *0.116). Additionally, when compared with non‐DEGs, they did not show a larger differentiation (as calculated using *F*_*ST*_ between eastern wild and cultivated accessions, Wilcoxon rank sum test; *P *>* *0.05) nor a decrease in nucleotide diversity, whether synonymous or non‐synonymous, in the cultivated accessions (Wilcoxon rank sum test; *P *>* *0.05).

## Discussion

Here, we studied the evolutionary history of the olive using transcriptomic data of both wild and cultivated accessions from all around the Mediterranean basin. Oleasters were carefully selected to represent genuinely wild olives not heavily impacted by human activities (Besnard and Rubio de Casas, 2016). Their wild origin was corroborated through their high diversity in cpDNA haplotypes and their genetic differentiation observed at the nuclear genome.

### Insights into the domestication history of olives

#### East/West geographic structure in olives and admixture in the cultivated gene pool

Expression data, transcriptomic variants and chloroplastic data all confirmed the previously described high East/West differentiation observed in olives (Besnard *et al*., 2013a; Díez *et al*., 2015), in line with the hypothesis of a geographic structure older than the domestication process (Besnard *et al*., 2013b). The diversity of cultivated olives also displayed an East/West structure, but in contrast with oleasters, there is extensive admixture between these two gene pools, as already evidenced (Besnard *et al*., 2013b; Díez *et al*., 2015; Khadari and El Bakkali, 2018).

Based on few markers, previous investigations identified two cultivated populations (namely eastern and western) (Besnard *et al*., 2001b), or three (namely western, central and eastern populations, Haouane *et al*., 2011; Belaj *et al*., 2012; Díez *et al*., 2012, 2015; El Bakkali *et al*., 2013). Our SNP dataset identified two major groups: cultivated accessions sampled in the East of the Mediterranean as only marginally differentiated from eastern oleasters in terms of nuclear diversity, and those from the West and Central Mediterranean basin that had mixed ancestry and were difficult to be further differentiated into separate populations.

#### Support for a single primary domestication in olives

We re‐examined the debate of single versus multiple domestication event(s) in olives, and several lines of evidence corroborate the occurrence of a dominant event in the eastern part of the Mediterranean basin followed by human‐mediated spread towards the West and introgression by western oleasters. First, there was weak differentiation between eastern oleasters and eastern cultivated accessions observed in both expression and SNPs data along with a slight reduction in diversity observed in the domesticated gene pool. Agronomically interesting oleasters may therefore have been put into cultivation in this region through cloning directly or after only a few generations of (conscious or unconscious) selection. Conversely, we could not identify any cultivar unambiguously assigned to the western group as they are all at least admixed between the two gene pools. Second, most cultivars (82%), both eastern and western, carried a chlorotype that is restricted to the eastern part of the Mediterranean basin in oleasters. This situation indicates that, for a majority of olive cultivars, the maternal origin can be traced to the East of the distribution. Thirdly, in the analysis of population split and mixtures, the western cultivars grouped with eastern oleasters and cultivated accessions rather than grouped with western oleasters, even when the western cultivated population was split into two populations. Lastly, western cultivated accessions are also closer to eastern wild and cultivated olives than to western oleasters in term of expression patterns. We therefore argue in favour of a single domestication event in olive in the eastern region of the Mediterranean basin as previously proposed (Besnard *et al*., 2013a; Khadari and El Bakkali, 2018). But we do recognize a secondary contribution of western oleasters in the cultivated gene pool of the western and central Mediterranean basin. Western cultivars indeed showed up to 60% of admixture from the western oleasters while a minority displayed a chlorotype only found in western oleasters (see Besnard *et al*., 2013b for a large sampling of cultivars from this area). During the expansion of olive cultivation to the West, domesticated olives were therefore repeatedly crossed with western oleasters.

This model of domestication, with introgression from wild relatives leading to cultivars closely related to their secondary progenitor, has already been observed in other perennials such as grape (Myles *et al*., 2011), apple (Cornille *et al*., 2012) and date palm (Gros‐Balthazard *et al*., 2017; Flowers *et al*., 2019).

Based on our dataset, we did not retrieve the scenario of independent domestication in the central Mediterranean basin favoured by Díez *et al*. (2015) who, based on microsatellite data, suggested a separate olive domestication in the central Mediterranean basin. However, their conclusion might have been driven in part by eastern oleaster accessions that turned out to be feral rather than genuinely wild (Díez *et al*., 2015; Díez and Gaut, 2016). Also, such an additional domestication, if it existed, must have been rather localized as it did not leave any detectable trace in neither the western nor eastern cultivars analyzed here.

### The consequence of domestication in the olive transcriptome

#### Reduction in nucleotide diversity but not in expression diversity

On average, cultivated olives retained 97% of the diversity found in oleasters. When only considering eastern populations, this number falls to 86% and 77% for nucleotide diversity (π) and the number of segregating sites (θ_*W*_), respectively. This deficit is comparable with that found in other perennials (94.6% on average; Miller and Gross, 2011). For instance, genomic data indicated that cultivated grapes retained about 95% of the diversity found in its wild progenitor (Zhou *et al*., 2017). This limited loss of diversity in perennials is related to the low number of generations since domestication and ongoing gene flow between wild populations and domesticates (Miller and Gross, 2011; Gaut *et al*., 2015). Interestingly, the western cultivars showed the largest nucleotide diversity, in line with their mixed ancestry including both eastern cultivated accessions and western oleasters. This high level of genetic diversity following a secondary contact of the cultivated gene pool with other wild relative populations has already been described in olives (Díez *et al*., 2015) and in other crops such as the grape vine (Myles *et al*., 2011) or the date palm (Gros‐Balthazard *et al*., 2017; Flowers *et al*., 2019).

In contrast with the nucleotide diversity, however, we did not find a significant reduction in the diversity of gene expression. While such a reduction is reported for annual crops such as rice (5.1% reduction; Liu *et al*., 2019) or common bean (18% reduction; Bellucci *et al*., 2014), it might be a result of the generally weaker domestication syndrome in perennial plants. However, an important limitation of our result is the restricted number of samples used for the expression analysis, and further observations might provide a more robust outcome.

#### Altered gene expression in cultivated olives

We found evidence of an important transcriptome rewiring due to domestication. Indeed, many transcripts exhibited differential expression when comparing wild and cultivated olives, whether using the full dataset, eastern olives or western olives separately. The observed fraction of DEGs linked to olive domestication (1.39%) is comparable with that found in maize (2.18%; Swanson‐Wagner *et al*., 2012) or in common bean (0.7%; Bellucci *et al*., 2014).

We identified six GO terms that were enriched in DEGs related to domestication. Of those, four were associated with transcriptional activity. This result is consistent with previous findings underlying an over‐representation of transcription regulators among domestication genes (Doebley *et al*., 2006; Meyer and Purugganan, 2013; Olsen and Wendel, 2013). We did not identify any DEGs directly related to oil content or fruit/seed size, traits expected to have been impacted by domestication. However, we note that the expression data analyzed here were obtained from leaves and inflorescences rather than fruits. Nevertheless, we provide evidence of olive transcriptome reshaping by domestication, motivating the expression profiling of other tissues, especially the fruit, to further understand how the olive transcriptome was affected.

#### Lack of signatures of selection in the olive transcriptome

Estimates of the extent to which artificial selection has shaped crop genomes mostly stem from annuals and include ~7.6% for maize (Hufford *et al*., 2012), ~9% for the common bean (Bellucci *et al*., 2014) and ~7.3% in sunflower (Chapman *et al*., 2008). Estimates are lower for the perennial apple, for which regions selected during the initial phase of domestication or secondary introgression covered only 3.7% of the genome (Duan *et al*., 2017).

Here we report even weaker selection on protein‐coding genes in olives. Both statistical approaches used (PCAdapt and BayeScan) to contrast eastern oleasters and eastern cultivated accessions failed to identify candidate genes for domestication, and only 10 (0.04%) putative selected genes were detected when using the full dataset. While our estimates are based on transcriptomes rather than full genomes, the very weak selection inferred here is a likely result of olive life history. Indeed, olives are self‐incompatible with long generation times, and are extensively propagated clonally while still exchanging genes with oleasters (Rugini *et al*., 2016), all limiting the possibility to impose strong selection regimes. But we note that our selection scans were underpowered to detect selection on highly polygenic traits (Narum and Hess, 2011) that were proposed to contribute substantially to the domestication in other crops (Olsen and Wendel, 2013). In addition, and given the long generation time, selection during domestication was likely to act primarily on standing variation rather than on *de novo* mutations (Stetter *et al*., 2017), therefore leaving even softer signatures of selection that are harder to detect (Przeworski *et al*., 2005).

#### The olive domestication syndrome driven by gene expression changes

In contrast with the weak signature of selection we found on coding genes, there was a much higher proportion of changes in gene expression potentially associated with olive domestication. While we detected only 0.04% of putative genes under selection, we found that 1.39% of these same genes were differentially expressed in wild and cultivated eastern olives. Several non‐exclusive hypotheses may explain how the expression patterns were rewired during olive domestication without leaving strong signatures of selection in the transcriptome. First, gene expression may have changed as a result of drift, in relation to the demography of olives, rather than artificial selection. Second, and given the polygenic nature of gene regulation with a large numbers of transcription factors modulating the expression of single genes (Chen and Rajewsky, 2007), our data set was underpowered to detect selection signals. Third, mutations in *cis*‐regulatory regions (CREs) played a major role in changing expression patterns. CREs are known to be major targets of evolutionary changes, due to their weaker deleterious pleiotropic effects compared with mutations in coding sequences (Stern and Orgogozo, 2008), as was exemplified in annual crops (Lemmon *et al*., 2014; Wang *et al*., 2017; but see Rhoné *et al*., 2017). Yet, they were not captured by our transcriptomic data and, in case of rapid decay of linkage disequilibrium, signatures of selection could not extend up to the coding regions we sequenced. Lastly, epigenetic changes, maintained through vegetative propagation (Richards, 2011), can also contribute to gene expression changes associated with domestication as was recently shown for agronomically important traits such as fruit ripening in tomato (for review, see Gallusci *et al*., 2016; Piperno, 2017). Interestingly, siRNA involved in epigenetic processes were recently discovered to be involved in oil biosynthesis in olive (Unver *et al*., 2017).

Phenotypic differences between wild and cultivated olives may therefore have emerged from divergent gene regulation rather than changes in protein function through fixed difference in amino acid sequence of structural genes (*aka* gene coding for any RNA or protein product other than a regulatory factor). This finding is consistent with the evolutionary history and the life history of olives. Indeed, in perennials, there has been a limited number of generations since the onset of artificial selection (due to long generation time and clonal propagation) and previous experimental evolution studies showed that the response to artificial selection occurs primarily through gene expression changes rather than changes of allele frequencies at coding sites (Konczal *et al*., 2015). Additionally, it was shown in steelhead trout (*Oncorhynchus mykiss*) that the expression of hundreds of genes was heritably altered after just a single generation of domestication (Christie *et al*., 2016). The weak domestication syndrome observed in olives therefore probably arose following heritable changes of gene regulation.

Although more challenging than studying annual crops, further studies of the genomic of the domestication process in perennial should broaden our understanding of the genomic bases and dynamics of adaptation, especially to understand whether they are influenced by the life history and ecological traits of a species.

## Experimental procedures

### Plant material

We analyzed a total of 68 accessions of *Olea europaea* L., representing 27 oleasters [*Olea europaea* subsp. *europaea* var. *sylvestris* (Mill.) Lehr], 39 cultivated olive trees (*Olea europaea* subsp. *europaea* var. *europaea*), and two accessions of African olive [*Olea europaea* subsp. *cuspidata* (Wall. ex G.Don) Cif.] (Table S1).

Eastern and western oleasters were sampled to represent the two wild gene pools previously evidenced (Besnard *et al*., 2001b, 2013b; Breton *et al*., 2006). We sampled 17 oleasters in the area south of the Taurus mountains (South Turkey near the border with Syria), a region considered as very close to the primary centre of olive domestication (Besnard *et al*., 2013b) (Table S1). Five additional eastern oleasters were sampled in the CBNMed collection at Porquerolles Island, France: two from Western Turkey, and three from Northwestern Syria. Five western oleasters were sampled in three different locations in Morocco, with one of these conserved at the CBNMed. To minimize admixture with cultivated olives, most of these wild forms were sampled in natural areas far from olive agroecosystems. The sampled oleasters displayed smaller fruits characterized by less fleshy mesocarp than cultivated olives; yet this characteristic may also be found in feral individuals (Hannachi *et al*., 2008). Therefore, we characterized their chloroplastic (cpDNA) variation (Methods S1 and Data S1) as true oleasters are expected to harbour a higher diversity of plastid profiles with many haplotypes never observed in cultivated olives (Besnard *et al*., 2013b).

The 39 cultivated accessions were sampled in three germplasm collections (Table S1) and were chosen to maximize the Mediterranean diversity (El Bakkali *et al*., 2013). Three pairs of accessions (OGMed_025/OGMed_026 from Cyprus, OGMed_029/OGMed_046 from Lebanon and Italy, and OGMed_018/OGMed_055 from Morocco) sharing the same nuclear microsatellite profile (El Bakkali *et al*., 2013) were considered as clones and used to calibrate the relatedness and differential gene expression analyses.

### RNA‐sequencing, read alignments and variant calling

We performed three sequencing runs in 2011, 2013 and 2015 to obtain a total of 68 *Olea* spp. transcriptomes (Table S1). Total RNA was extracted from leaves and/or inflorescence tissues. The construction of Illumina libraries and sequencing protocols are described in Sarah *et al*., (2017). The data obtained for the cultivar ‘Arbequina’ and on a set of 10 wild olive trees and for one accession *O. e. cuspidata* were previously used by Sarah *et al*. (2017) and Clément *et al*. (2017) (see Table S1).

We used a custom bioinformatic pipeline, following in large the standard Illumina pipeline, to process raw reads, obtain alignment files and generate variant call files (Figure S15 and Methods S2). In brief, raw Illumina reads were filtered based on quality before mapping on the annotated olive transcriptome assembly of var. ‘Arbequina’ (Sarah *et al*., 2017) with BWA (Li and Durbin, 2010). Keeping only high quality alignments, we performed local realignment near InDels using GATK (McKenna *et al*., 2010). Variants were called using GATK and filtered following different criteria (Methods S2).

### Genetic structure and diversity in wild and cultivated olives

We first inferred relatedness between each pair of olive accessions (Methods S3). We estimated individual ancestries using NGSadmix (Skotte *et al*., 2013) on all olive accessions and on wild and cultivated accessions separately with a minimum minor allele frequency of 0.05. Input files were directly generated from alignment files with ANGSD v.0.911 (Korneliussen *et al*., 2014) using the genotype likelihood estimation as implemented in SAMtools (Li *et al*., 2009). A PCA was performed using genotype calls as input and considering a minimum minor allele frequency of 0.05 with the PCAdapt *R* package v.3.0.2 (Duforet‐Frebourg *et al*., 2014, 2015; Luu *et al*., 2017). We used TreeMix v.1.12 (Pickrell and Pritchard, 2012) to discriminate trees between wild and cultivated populations, and identify gene flows between them.

Genetic variation within *O. europaea* was assessed using different statistics. We calculated observed and expected heterozygosities (*H*_*O*_ and *H*_*E*_), the mean number of pairwise differences π (Tajima, 1983), the Watterson estimator θ_*W*_ (Watterson, 1975), and Tajima's *D* (Tajima, 1989) based on site frequency spectra (SFSs) inferred with ANGSD v.0.911 (Korneliussen *et al*., 2014), as detailed in Methods S4. These statistics were calculated for all sites and on both synonymous and non‐synonymous sites annotated as described in Methods S5. In addition, the partitioning of diversity within and between populations, measured with *F*_*ST*_ (Weir and Cockerham, 1984), was calculated using VCFtools (Danecek *et al*., 2011).

### Gene expression in wild and cultivated olives

To avoid issues related to the independent construction of libraries (Conesa *et al*., 2016), we focused on the first sequencing run, as it contained data from all four populations studied here (Table S1). We therefore included five oleasters from both eastern and western Mediterranean basin, along with three and six accessions of eastern and western cultivated olives, respectively. Read counts (the number of read pairs aligned to each transcript) were calculated using the *idxstats* option of SAMtools. We excluded genes with very low expression levels (< 30 mapped reads across all 19 sequenced libraries) and high within‐group variance (> 100 000 in wild or cultivated populations). After data normalization using the *DESeq2* package from Bioconductor (Anders and Huber, 2010; Love *et al*., 2014), we clustered gene expression hierarchically using *hclust* in *R* software (R Core Team, 2015). We inferred gene expression diversity for each sample by calculating the coefficient of variation (standard deviation divided by the mean) of the expression values (Bellucci *et al*., 2014). We tested for differences in gene expression diversity between populations using Student's *t*‐tests. We then identified DEGs with *DESeq2* which fits a generalized linear model (GLM) modelling read counts per gene and samples using a negative binomial distribution. *P*‐values were computed using a Wald test (Love *et al*., 2014) and transformed into *q*‐values to correct for multiple testing using the *R* package *qvalue* (Storey, 2002; Bass *et al*., 2015). Genes were considered as differentially expressed if they showed a *q*‐value ≤ 0.01.

GO terms enriched in differentially expressed transcripts (functionally annotated by Sarah *et al*., 2017) were established with the *R* package *goseq* (Young *et al*., 2010), using a Wallenius distribution as null distribution and a false‐discovery threshold of 0.05 (Benjamini and Hochberg, 1995).

### Genes under selection during domestication

We first measured the strength of the selective constraint *C *=* *1 – (π_*NS*_/π_*S*_) (Keightley *et al*., 2011, also known as evolutionary constraint) from the diversity at non‐synonymous (π_*NS*_) and synonymous sites (π_*S*_), assuming the latter to be neutral.

To minimize false positives, we accounted for the demographic history of olives when identifying molecular signatures of selection using two complementary approaches (Lotterhos and Whitlock, 2014; Vatsiou *et al*., 2016). First, we used a Bayesian approach implemented in BayeScan v.2.1 (Foll and Gaggiotti, 2008) to detect SNPs with unusually high *F*_*ST*_ values, which is a hallmark of divergent selection. This analysis was performed on different sets of populations and we ran BayeScan twice for each model with prior odds of the neutral model set to 100 and 1000, respectively. We used an initial burn‐in period of 5000 steps. After 20 pilot runs of 5000 iterations each, we sampled 5000 parameter combinations with a thinning interval of 10, resulting in a total of 100 000 iterations. The probability that a locus is under selection was estimated by calculating the Bayes factor of a model with selection over a neutral model and loci with a *q*‐value < 0.05 were considered as potential candidates for selection. Second, and because the presence of admixed individuals may impact the power of BayeScan (Luu *et al*., 2017), we also identified candidate SNPs using PCAdapt v2.2.1, a hierarchical Bayesian approach (Duforet‐Frebourg *et al*., 2014, 2015). PCAdapt first performs a PCA to infer population structure and then identifies loci that are atypically related to a specific principal component as measured by the latent factors. We corrected for multiple testing by transforming *p*‐values associated with each locus into *q*‐values (Storey, 2002) using the *R* package *qvalue* v.2.4.2 (Bass *et al*., 2015) and used a false‐discovery threshold of 0.01.

To gain a deeper biological insight into the candidate SNPs retrieved from these selection scans, we performed GO enrichment analysis using the *R* package SNP2GO version 1.0 (Szkiba *et al*., 2014) using default parameters. The lists of enriched GO terms were summarized and visualized using REVIGO (Supek *et al*., 2011).

Finally, we tested whether identified DEGs are more frequently identified as targets of selection than non‐DEGs by comparing their *F*_*ST*_ and π values using Wilcoxon rank sum tests.

## Accession numbers

The data were deposited in the Sequence Read Archive (SRA) under the Bioproject accession PRJNA525000.

## Conflict of interest

The authors declare no conflict of interest.

## Author contributions

MG‐B, GB, SG and BK designed the research. MG‐B performed data analysis and interpretation and wrote the manuscript with the help of GB, DW, SG and BK. GB, SG YH, JL, SS, DW, SG and BK contributed to data analysis and interpretation. BK collected the material.

## Supporting information


**Figure S1.** Heatmap displaying the relatedness between the 66 Mediterranean olive tree accessions.
**Figure S2.** Admixture proportions in 62 olive trees inferred from genotype likelihoods of 536 341 SNPs with NGSadmix for *K *=* *4–6.
**Figure S3.** Admixture proportions inferred from genotype likelihoods with NGSadmix for *K* = 2–6.
**Figure S4.** Log‐likelihood estimates for different *K* values in the three admixture analyses.
**Figure S5.** Proportion of variance explained by the 10 first principal components of the PCA performed on 62 olive tree accessions.
**Figure S6.** Principal component analysis of 62 *Olea europaea* subsp. *europaea*.
**Figure S7.** Reduced median network of chlorotypes reconstructed with network v.5 using data from Data S1.
**Figure S8.** Fraction of variance in relatedness between four olive tree populations accounted for by phylogenetic models with zero through two migrations in TreeMix.
**Figure S9.** TreeMix relationships between oleasters and cultivars from all around the Mediterranean basin grouped in four populations.
**Figure S10.** TreeMix relationships between five olive tree populations.
**Figure S11.** Fraction of variance in relatedness between five olive tree populations accounted for by phylogenetic models with zero through two migrations in TreeMix.
**Figure S12.** Bayesian test for selection between 27 wild and 36 cultivated populations implemented in BayeScan.
**Figure S13.** Principal component analysis of SNPs data.
**Figure S14.** Visualizations of enriched GO terms from PCAdapt selection scan including all accessions.
**Figure S15.** Bioinformatic pipeline followed to process the raw reads into alignment files (final*.bam* files) and subsequently variant call format file (.*vcf* file) used in subsequent analyses.

**Table S1.** Summary of the 68 *Olea europaea* spp. accessions including their origin, the cultivar name when relevant and statistics related to sequencing (excel file).

**Table S2.** List of SNPs detected as outliers in BayeScan when comparing all oleasters and all cultivated accessions.
**Table S3.** Strong candidate transcripts for selection.

**Methods S1.** Plastid genome profiling.
**Methods S2.** Read alignments and variant calling.
**Methods S3.** Inference of relatedness among accessions.
**Methods S4.** Inference of site frequency spectra and calculation of diversity statistics.
**Methods S5.** Annotation of the transcriptome reference sites as synonymous or non‐synonymous.

**Data S1.** Chloroplastic microsatellite and CAPS profile of each individual analysed in the present study [according to the method developed by Besnard *et al*. (2011) (excel file).

**Appendix S1.** Detailed structure and diversity of oleaster populations.
