# Supplementary material for: Evolutionary transcriptomics reveals the origins of olives and the genomic changes associated with their domestication
Source: Plant J. 2019 Jul 11;100(1):143–57. doi: 10.1111/tpj.14435 (PMC6851578; doi:10.1111/tpj.14435)
Supplement: Supplementary file 1 — Figure S1. Heatmap displaying the relatedness between the 66 Mediterranean olive tree accessions. Figure S2. Admixture proportions in 62 olive trees inferred from genotype likelihoods of 536 341 SNPs with NGSadmix for K = 4–6. Figure S3. Admixture proportions inferred from genotype likelihoods with NGSadmix for K = 2–6. Figure S4. Log‐likelihood estimates for different K values in the three admixture analyses. Figure S5. Proportion of variance explained by the 10 first principal components of the PCA performed on 62 olive tree accessions. Figure S6. Principal component analysis of 62 Olea europaea subsp. europaea. Figure S7. Reduced median network of chlorotypes reconstructed with network v.5 using data from Data S1. Figure S8. Fraction of variance in relatedness between four olive tree populations accounted for by phylogenetic models with zero through two migrations in TreeMix. Figure S9. TreeMix relationships between oleasters and cultivars from all around the Mediterranean basin grouped in four populations. Figure S10. TreeMix relationships between five olive tree populations. Figure S11. Fraction of variance in relatedness between five olive tree populations accounted for by phylogenetic models with zero through two migrations in TreeMix. Figure S12. Bayesian test for selection between 27 wild and 36 cultivated populations implemented in BayeScan. Figure S13. Principal component analysis of SNPs data. Figure S14. Visualizations of enriched GO terms from PCAdapt selection scan including all accessions. Figure S15. Bioinformatic pipeline followed to process the raw reads into alignment files (final.bam files) and subsequently variant call format file (.vcf file) used in subsequent analyses. [file TPJ-100-143-s001.docx]

**Fig. S1 Heatmap displaying the relatedness between the 66 Mediterranean olive tree accessions.** Relatedness was calculated as explained in Supporting Information Methods S3.

**Fig. S2 Admixture proportions in 62 olive trees inferred from genotype likelihoods of 536,341 SNPs with NGSadmix for *K* = 4 to 6.** On the top of the graph, colored dots indicate the chloroplastic lineage of each accession. Grey: E1; brown: E2; blue: E3; white: L1 (Table S2). WO: western oleasters; WC: western cultivars; EC: eastern cultivars; EO: eastern oleasters.

**Fig. S3** **Admixture proportions inferred from genotype likelihoods with NGSadmix for *K* = 2 to 6.** (a) 35 cultivated olive trees (WC: western cultivars; EC: eastern cultivars), and (b) 27 wild olive trees (WO: western oleasters; EO: eastern oleasters). Horizontal black lines indicate the different populations of eastern oleasters.

**Fig. S4** **Log-likelihood estimates for different *K* values in the three admixture analyses.** Left: log-likelihood change for increasing number of *K*; right: Change in log-likelihood for increasing number of *K*. (a) Main analysis comprising 62 wild and cultivated olives; (b) Analysis comprising 35 cultivated olives; (c) Analysis comprising 27 oleasters.

**Fig. S5** **Proportion of variance explained by the ten first principal components of the PCA performed on 62 olive tree accessions.**

**Fig. S6** **Principal Component Analysis of 62 *Olea europaea* subsp. *europaea*.** EO: eastern oleasters; WO: western oleasters; EC: eastern cultivated olives; WC: western cultivated olives.


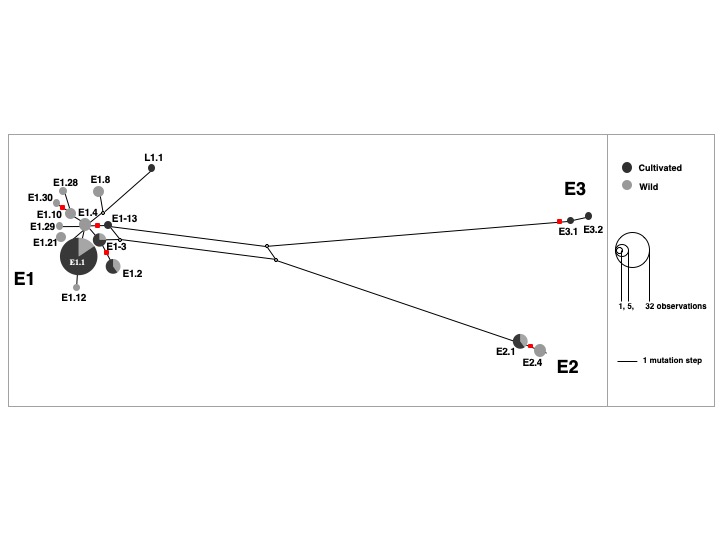


**Fig. S7** **Reduced median network of chlorotypes reconstructed with Network v.5 using data from Table S2.** The locus CAPS-*Xap*-I was excluded from the analysis because was reported as highly homoplasious (Besnard *et al.*, 2011); changes at this locus are represented by additional red squares on the network. The tree main Mediterranean maternal lineages E1, E2 and E3 are recognized as previously reported (Besnard *et al.*, 2013b). Note the placement of haplotype L1.1 (from Hoggar) sister to lineage E1.

**Fig. S8** **Fraction of variance in relatedness between four olive tree populations accounted for by phylogenetic models with zero through two migrations in TreeMix.** The dashed line corresponds to the cut-off value of 0.99. The addition of one event of migration greatly increases the variance explained while the addition of a second migration event does not increase it leading us to conclude that the relationship between the four olive tree populations is accurately described with a single migration edge.

**Fig. S9** **TreeMix relationships between oleasters and cultivars from all around the Mediterranean basin grouped in four populations.** (a) Model without migration and corresponding residuals on the right; (b) Model with two migration events and corresponding residuals on the right. The model with one migration event can be found in the main text (Fig. 3). OUT: outgroup *O. e. cuspidata* accessions; EO: eastern oleasters; EC: eastern cultivars; WC: western cultivars; WO: eastern oleasters.

**Fig. S10** **TreeMix relationships between five olive tree populations.** Compared to the model with four populations, western cultivated olives were split into two populations based on the result of the admixture analyses with *K* = 3 (Fig. 1). (a), (b) and (c) display models with 0, 1, and 2 migration events, respectively, with corresponding residuals on the right. OUT: outgroup *O. e. cuspidata* accessions; WO: western oleasters; EC: eastern cultivars; WC1: western cultivars not admixed with western oleasters; WC2: western cultivars admixed with western oleasters; WO: eastern oleasters.

**Fig. S11** **Fraction of variance in relatedness between five olive tree populations accounted for by phylogenetic models with zero through two migrations in TreeMix.** The dashed line corresponds to the cut-off value of 0.99. The addition of one event of migration greatly increases the variance explained while the addition of a second or third migration event does not increase it significantly, leading us to conclude that the relationship between the five olive tree populations is accurately described with a single migration edge.


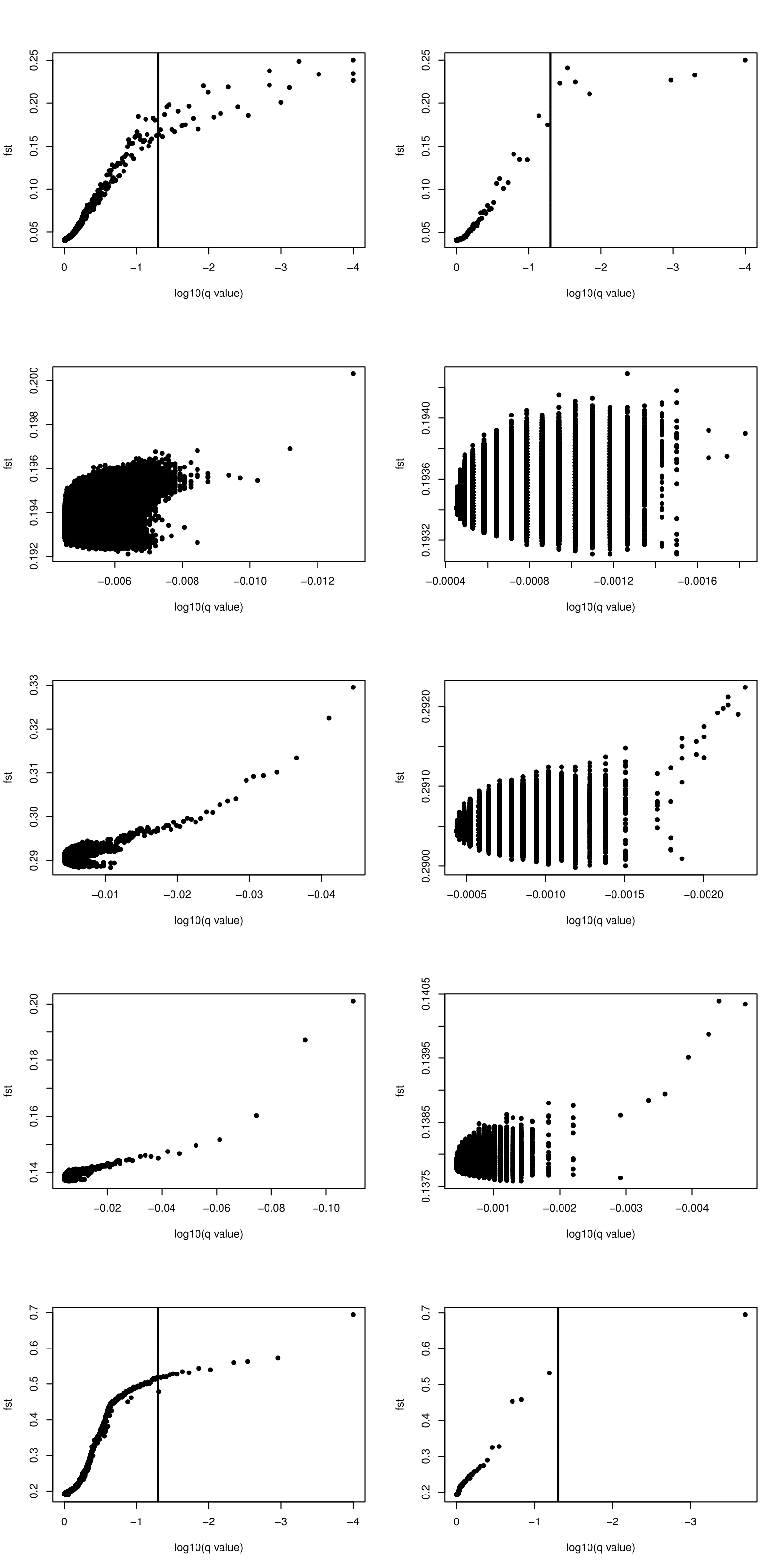


**Fig. S12 Bayesian test for selection between 27 wild and 36 cultivated populations implemented in BayeScan.** Outliers were detected by comparing different populations and according to different levels of detection indicated by vertical lines. Left: prior odds= 100; right: prior odds= 1,000.

**Fig. S13** **Principal Component Analysis (PCA) of SNPs data.** (a) 30 eastern olive accessions and (b) 32 western olive accessions. EO: eastern oleasters; EC: eastern cultivated olives; WO: western oleasters; WC: western cultivated olives.

(a)


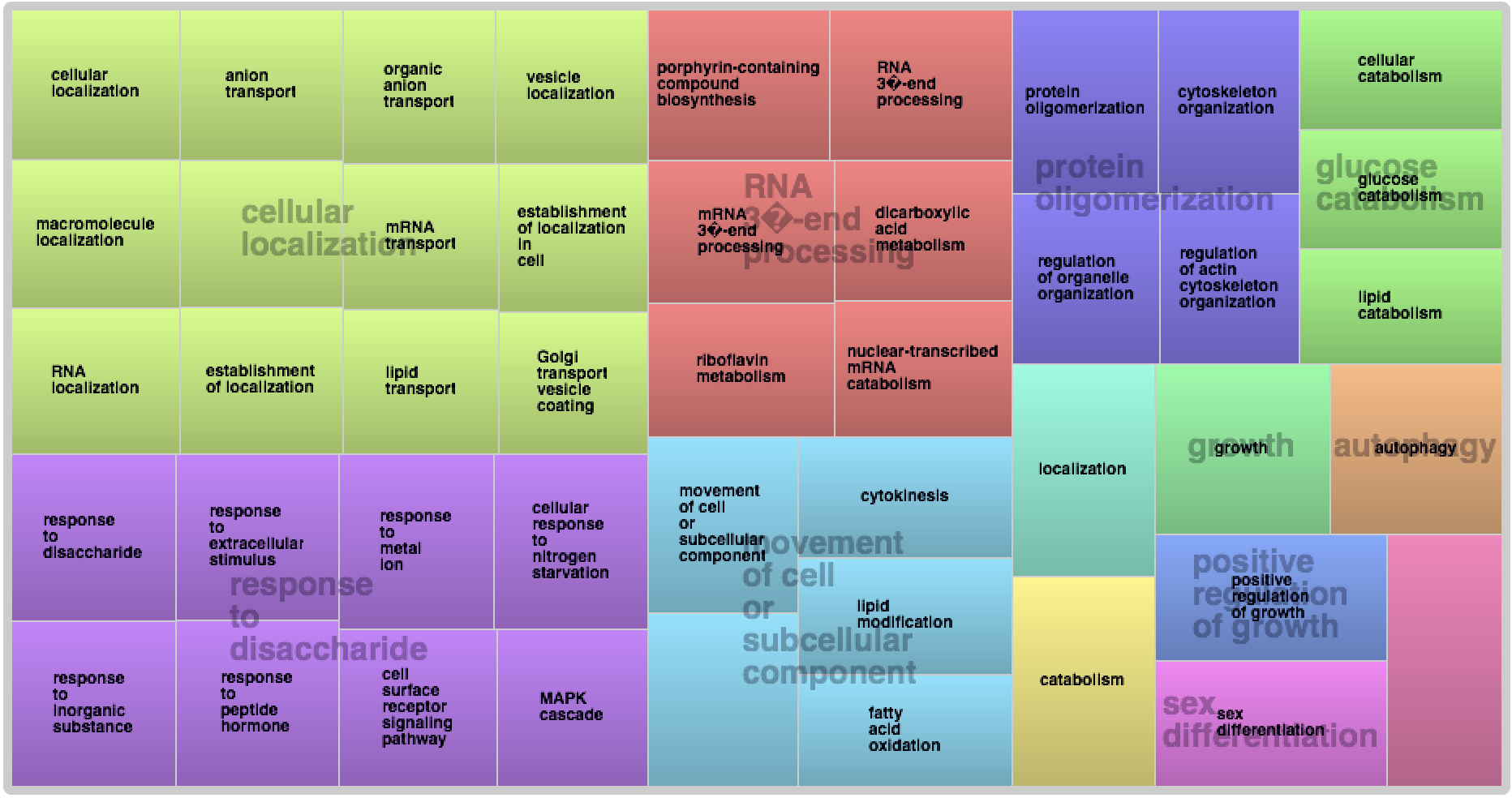


(b)


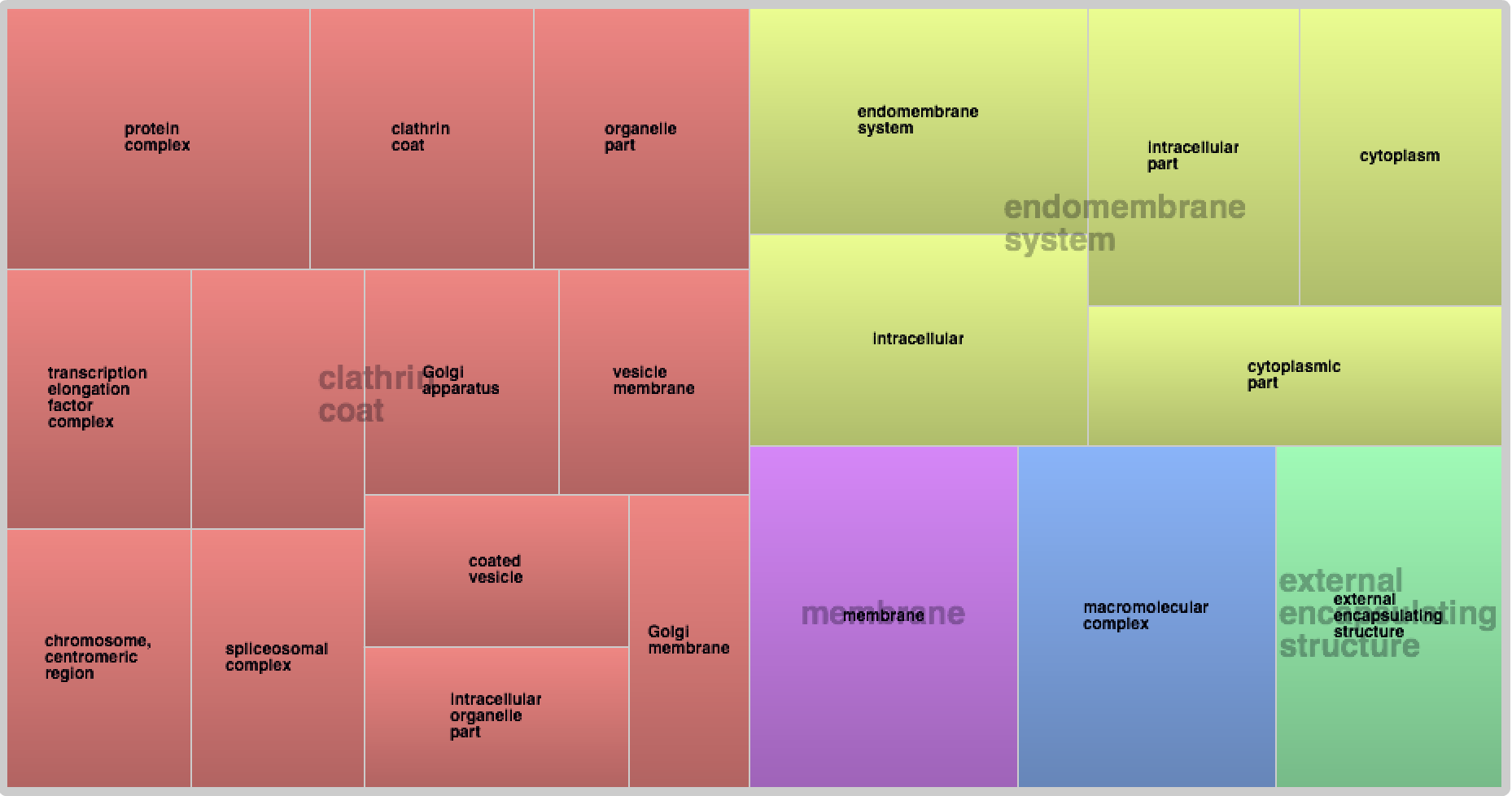


(c)


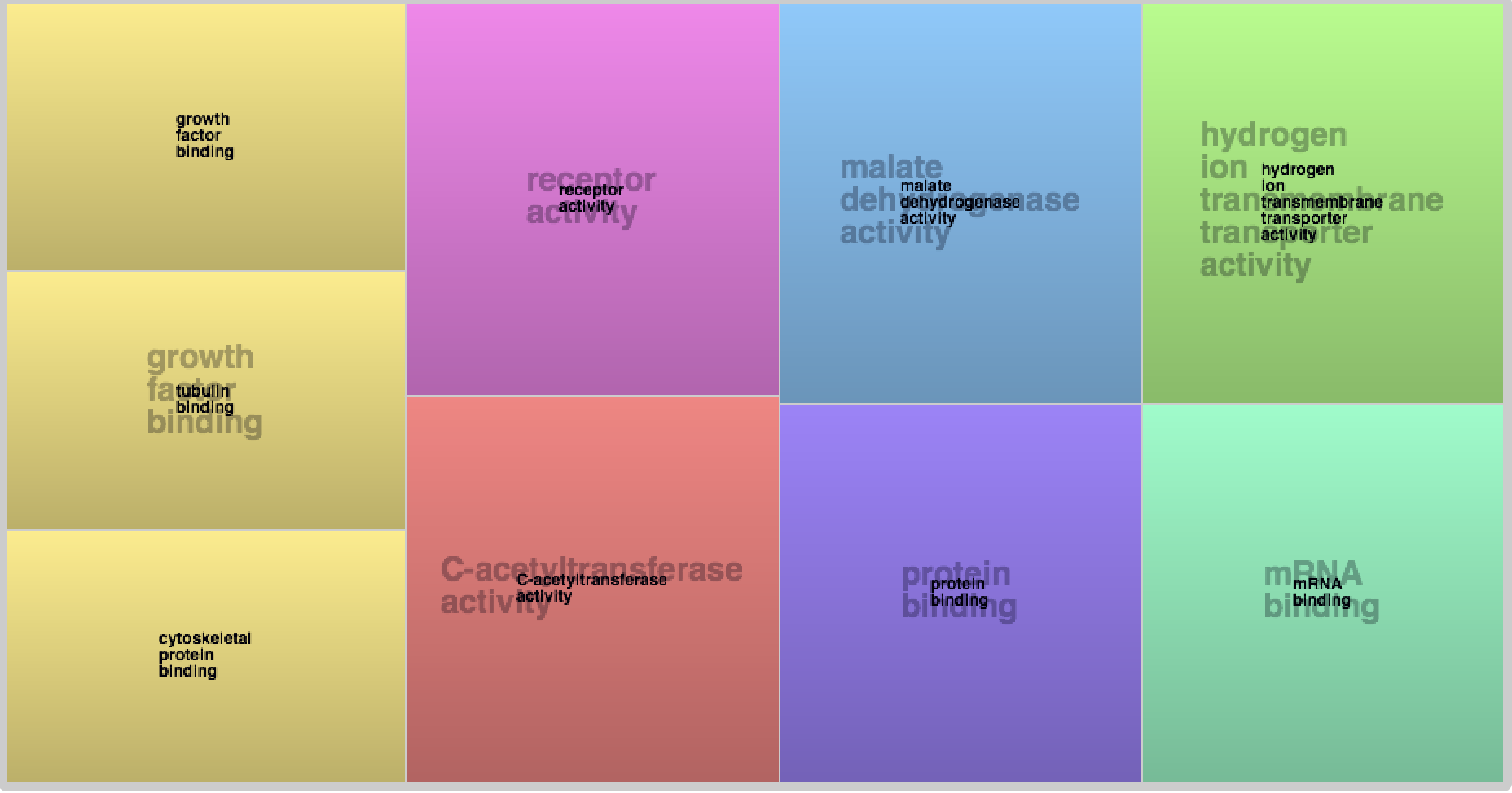


**Fig. S14** **Visualizations of enriched GO terms from PCAdapt selection scan including all accessions.** Ninety-seven GO terms were found significantly enriched (*P* < 0.05) among the top 1% outliers SNPs on the factor 2 of the PCA. (a) Biological process, (b) Cellular component, (c) Molecular function.


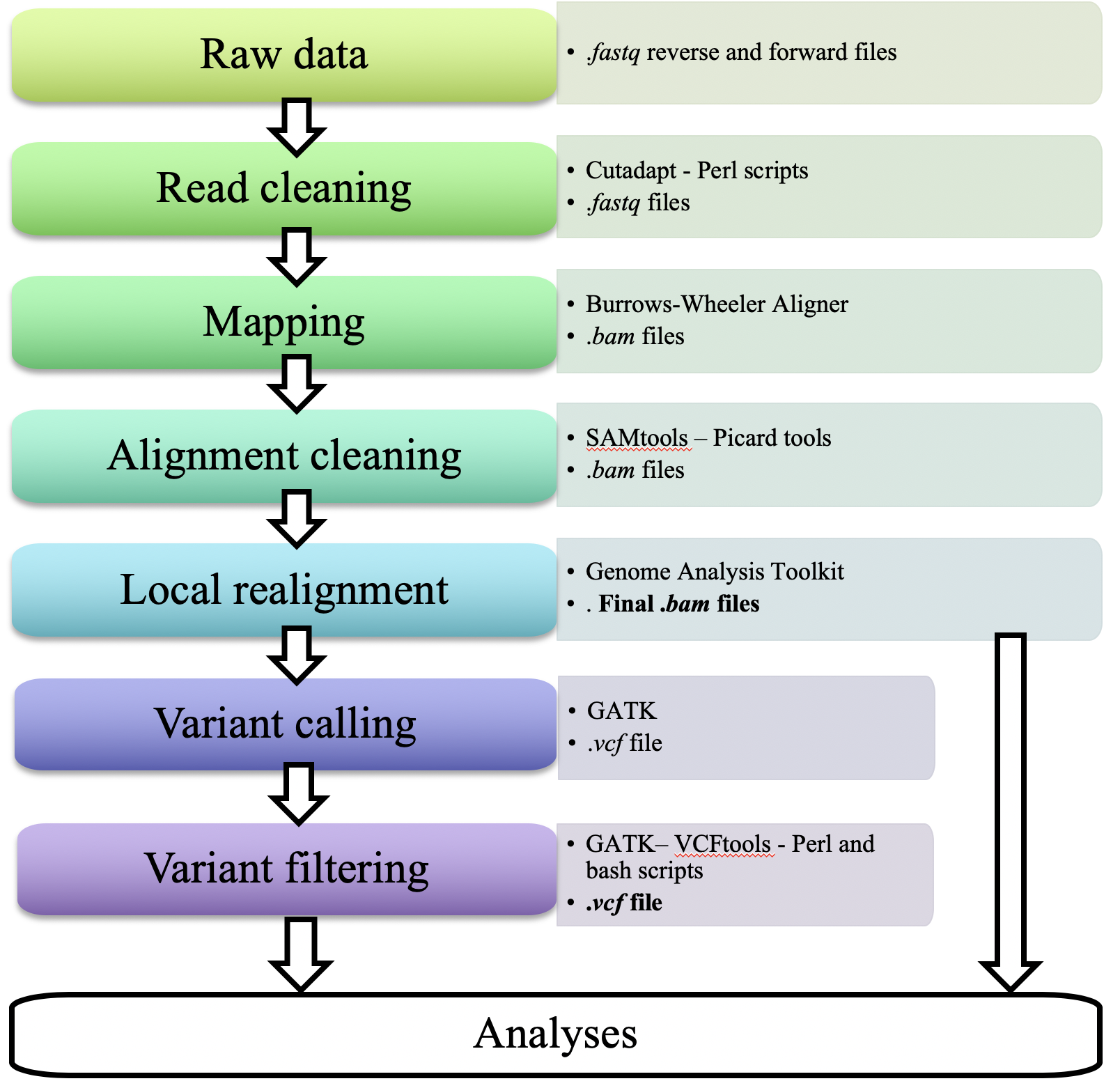


**Fig. S15** **Bioinformatic pipeline followed to process the raw reads into alignment files (final *.bam* files) and subsequently variant call format file (.*vcf* file) used in subsequent analyses.**
