# Supplementary material for: Evolutionary transcriptomics reveals the origins of olives and the genomic changes associated with their domestication
Source: Plant J. 2019 Jul 11;100(1):143–57. doi: 10.1111/tpj.14435 (PMC6851578; doi:10.1111/tpj.14435)
Supplement: Supplementary file 3 — Table S2. List of SNPs detected as outliers in BayeScan when comparing all oleasters and all cultivated accessions. Table S3. Strong candidate transcripts for selection. [file TPJ-100-143-s003.docx]

**Table S1** **Summary of the 68 *Olea europaea* spp. accessions including their origin, the cultivar name when relevant and statistics related to sequencing** (excel file).

**Table S2** **List of SNPs detected as outliers in BayeScan when comparing all oleasters and all cultivated accessions.** The two last columns indicate whether these SBPs where identified in the analysis with a prior odds (PO) of 100 or 1000.

| **Contig** | **Position** | **Prob** | **log10(PO)** | **q-value** | **Alpha** | **FST** | **PO 100** | **PO 1000** |
| --- | --- | --- | --- | --- | --- | --- | --- | --- |
| Contig17549 | 253 | 1 | 1000 | 0 | 2.1009 | 0.23447 | X | X |
| Contig17817 | 74 | 1 | 1000 | 0 | 2.0452 | 0.22649 | X | X |
| singlet__33392 | 244 | 1 | 1000 | 0 | 2.2112 | 0.2501 | X | X |
| singlet__252820 | 441 | 0.9988 | 2.9202 | 0.00030006 | 2.0839 | 0.23373 | X | X |
| Contig16096 | 422 | 0.9984 | 2.7951 | 0.00056011 | 2.1834 | 0.2486 | X | X |
| Contig18536 | 774 | 0.9982 | 2.7439 | 0.00076682 | 1.9779 | 0.21835 | X | X |
| Contig9773 | 421 | 0.997 | 2.5215 | 0.0014447 | 2.1108 | 0.23784 | X | X |
| singlet__4168 | 952 | 0.9976 | 2.6187 | 0.0010002 | 1.8533 | 0.20084 |  | X |
| singlet__16594 | 141 | 0.997 | 2.5215 | 0.0014447 | 1.9904 | 0.22102 |  | X |
| Contig7184 | 791 | 0.9848 | 1.8114 | 0.0028206 | 1.73 | 0.18583 |  | X |
| Contig7184 | 736 | 0.9846 | 1.8057 | 0.0039644 | 1.8007 | 0.19565 |  | X |
| Contig6926 | 479 | 0.9794 | 1.677 | 0.0053511 | 1.9614 | 0.21909 |  | X |
| Contig7184 | 840 | 0.9752 | 1.5946 | 0.0068475 | 1.7423 | 0.18814 |  | X |
| Contig7184 | 808 | 0.96999 | 1.5096 | 0.0085017 | 1.7059 | 0.18378 |  | X |
| singlet__214854 | 36 | 0.96599 | 1.4534 | 0.010202 | 1.9103 | 0.21302 |  | X |
| Contig16096 | 340 | 0.96399 | 1.4277 | 0.011815 | 1.9604 | 0.22036 |  | X |
| singlet__65590 | 281 | 0.95179 | 1.2954 | 0.013956 | 1.5815 | 0.16973 |  | X |
| Contig18536 | 765 | 0.94279 | 1.2169 | 0.016359 | 1.6746 | 0.1824 |  | X |
| singlet__250739 | 678 | 0.93719 | 1.1738 | 0.018804 | 1.7734 | 0.19637 |  | X |
| singlet__260923 | 871 | 0.93439 | 1.1535 | 0.021144 | 1.615 | 0.17489 |  | X |
| singlet__4168 | 710 | 0.93079 | 1.1287 | 0.023433 | 1.6013 | 0.17369 |  | X |
| Contig17126 | 1360 | 0.90998 | 1.0047 | 0.02646 | 1.7107 | 0.19062 |  | X |
| Contig11253 | 496 | 0.90158 | 0.96192 | 0.029589 | 1.5323 | 0.16676 |  | X |
| singlet__260923 | 1086 | 0.90058 | 0.95705 | 0.032498 | 1.5501 | 0.16934 |  | X |
| Contig9321 | 1255 | 0.89898 | 0.94934 | 0.035239 | 1.7616 | 0.19811 |  | X |
| singlet__269147 | 53 | 0.89218 | 0.91775 | 0.038031 | 1.7335 | 0.19575 |  | X |
| Contig7711 | 249 | 0.88318 | 0.87852 | 0.040949 | 1.6637 | 0.18686 |  | X |
| singlet__57906 | 130 | 0.87978 | 0.86438 | 0.04378 | 1.4735 | 0.16109 |  | X |
| Contig15788 | 652 | 0.87518 | 0.84579 | 0.046575 | 1.5277 | 0.16893 |  | X |
| singlet__4168 | 550 | 0.87057 | 0.82778 | 0.049337 | 1.4861 | 0.16373 |  | X |

**Table S3** **Strong candidate transcripts for selection.** These ten transcripts were among the top hits in both BayeScan and PCAdapt (Table 3). Their sequence similarity to NCBI database was assessed using blastn. The top blast hit is presented here along with blastn statistics.

| **Transcript** | **Top *blastn* hit** | **NCBI Reference sequence accession** | **Query cover** | **E value** | **Ident** | **Function (Reference)** |
| --- | --- | --- | --- | --- | --- | --- |
| Contig7184 | *Olea europaea* var. *sylvestris* transmembrane protein 53-B-like (LOC111395409), mRNA | XM_023021386.1 | 100% | 0 | 96% | Involved in cell cycle (Vogelstein *et al.*, 2000) |
| Contig11253 | *Olea europaea* var. sylvestris THO complex subunit 4D-like (LOC111369528), mRNA | XM_022991084.1 | 100% | 0 | 100% | Involved during transcription (Piruat & Aguilera, 1998) |
| Contig17549 | *Olea europaea* var. *sylvestris* cytochrome c oxidase subunit 6a, mitochondrial-like (LOC111410855), transcript variant X2, mRNA | XM_023041430.1 | 65% | 0 | 96% | Involved in ATP synthesis (Wikstrom *et al.*, 1981) |
| Contig17817 | *Olea europaea* var. *sylvestris* coiled-coil domain-containing protein 86 (LOC111396887), mRNA | XM_023023483.1 | 99% | 0 | 99% | RNA binding (The UniProt Consortium, 2014) |
| Contig18536 | *Olea europaea* var. *sylvestris* caffeoylshikimate esterase-like (LOC111388441), mRNA | XM_023013143.1 | 100% | 0 | 99% | Lignin biosynthetic pathway (Vanholme *et al.*, 2013) |
| singlet__4168 | *Olea europaea* var. *sylvestris* transmembrane protein 53-B-like (LOC111395409), mRNA | XM_023021386.1 | 100% | 0 | 100% | Involved in cell cycle (Vogelstein *et al.*, 2000) |
| singlet__33392 | No significant similarity found | / | / | / | / | / |
| singlet__65590 | *Olea europaea* var. *sylvestris* uncharacterized LOC111403778 (LOC111403778), mRNA | XM_023032399.1 | 100% | 0 | 99% | / |
| singlet__250739 | *Olea europaea* var. *sylvestris* uncharacterized LOC111388646 (LOC111388646), transcript variant X1, mRNA | XM_023013404.1 | 100% | 0 | 100% | / |
| singlet__269147 | *Olea europaea* var. *sylvestris* 40S ribosomal protein S11-3-like (LOC111383964), mRNA | XM_023008161.1 | 66% | 0 | 100% | Translation (The UniProt Consortium, 2014) |
