# Supplementary material for: Evolutionary transcriptomics reveals the origins of olives and the genomic changes associated with their domestication
Source: Plant J. 2019 Jul 11;100(1):143–57. doi: 10.1111/tpj.14435 (PMC6851578; doi:10.1111/tpj.14435)
Supplement: Supplementary file 4 — Methods S1. Plastid genome profiling. Methods S2. Read alignments and variant calling. Methods S3. Inference of relatedness among accessions. Methods S4. Inference of site frequency spectra and calculation of diversity statistics. Methods S5. Annotation of the transcriptome reference sites as synonymous or non‐synonymous. [file TPJ-100-143-s004.docx]

**Methods S1** **Plastid genome profiling.**

Chloroplasts are maternally inherited in olives and polymorphism of their genome has thus been used for phylogeographic analyses, in particular for tracing cultivated olive origins (Besnard *et al.*, 2011, 2013b). The chloroplast DNA (cpDNA) variation was characterized using the 39 loci that are polymorphic in the Mediterranean olive tree (protocol described by Besnard *et al.*, 2011, 2013b). These correspond to 34 microsatellites (Loci 1, 2, 6, 9, 11, 15, 17, 19, 21, 22, 23, 24, 25, 27, 28, 29, 31, 33, 36, 38, 39, 41, 46, 47, 48, 49, 50, 51, 52, 53, 54, 56, 57, 58, 59), three indels (10, 52, 61) and two single nucleotide polymorphisms (CAPS-*Eco*RI, CAPS-*Xap*I) (Table S2). For each individual, the combination of alleles at the 39 loci allowed defining a haplotype profile (or chlorotype) that was assigned to one of the four lineages detected in the Mediterranean olive (i.e. E1, E2, E3 and L1; see Besnard *et al.*, 2013a,b). The haplotypes were coded following Besnard *et al.* (2013b). A haplotype network was reconstructed with Network v.5 (Bandelt *et al.*, 1999) as previously described by Besnard *et al.*, (2011, 2013b).

Eighteen distinct chloroplast haplotypes were detected on the 66 *O. e. europaea* accessions (Table S2). These chlorotypes belong to lineages E1, E2, E3 or L1 (Figs. 2 and S8). The 27 oleasters display 13 different haplotypes. All eastern oleasters harbor a chlorotype belonging to lineage E1. Three chlorotypes were not detected in a previous study on 1,797 Mediterranean genotypes (Besnard *et al.*, 2013b) and were named E1.28, E1.29 and E1.30 (Table S2). The five western oleasters show chlorotypes of lineage E2. This is consistent with previous analyses showing that western and central oleasters display chlorotypes from either E2 or E3 lineages (Besnard *et al.*, 2013b). Besides, the 39 accessions of cultivated olive display nine different chlorotypes: 33 bear a chlorotype of lineage E1 (84.6%), three of lineage E2 (7.7%), two of lineage E3 (5.1%) and one of lineage L1 (2.6%).

Among the 18 chlorotypes, five were private to the cultivated accessions (i.e., E1.13, E2-3, E3-1, E3-2, L1.1). Yet, among those five, four were already observed in oleasters (Besnard *et al.*, 2013a), while L1.1 originates from the Laperrine's olive, that occurs in the Saharan Mountains (Besnard *et al.*, 2013a). In contrast, nine chlorotypes were here private to oleasters (i.e. E1.4, E1.8, E1.10, E1.12, E1.21, E1.28, E1.29, E1.30, E2.4), with seven of them never observed in cultivated olives (i.e. E1.4, E1.8, E1.12, E1.21, E1.28, E1.29, E1.30; Besnard *et al.*, 2013a). This strengthens the hypothesis that these oleasters are genuinely wild rather than feral.

**Methods S2 Read alignments and variant calling.**

Raw Illumina reads were trimmed with a quality parameter of 20 applied to each base and adapters were removed using Cutadapt 1.8 (Martin, 2011)⁠⁠. Reads with a mean quality below 30 and a length below 35 bp were filtered out and only paired reads were kept so that 82.8% of the reads were finally proceeded for mapping.

We used the olive transcriptome sequence of var. 'Arbequina' (Sarah *et al.*, 2017) as a reference for mapping. This transcriptome reference is composed of 45,389 contigs of size ranging from 250 to 15,578 bp, with an average of 794 bp. We assessed its quality by evaluating the presence of 248 core eukaryotic genes using CEGMA (Parra *et al.*, 2007). Overall, 85.08 % were found complete and this number reached 95.97 % when counting the partial genes indicating the high quality of the transcriptome sequence. Cleaned reads of our samples were then aligned against this reference transcriptome with the Burrows-Wheeler Alignment (BWA, version 0.7.12-r1039; Li & Durbin, 2010) using the *bwa* algorithm and the option to remove duplicates. On average, 64.2% of reads mapped to our reference. More precisely, there were 60.1% and 67.0% of reads mapping the reference for oleasters and cultivated accessions, respectively (Table S1). The largest mapping rate observed in cultivated accessions might be related to the alignment on a transcriptome assembly generated from a cultivar, thus more closely related to cultivated accessions than oleasters. Alignments were converted to binary format and cleaned using SAMtools version 1.2 (Li *et al.*, 2009) following the subsequent criteria: discard reads with >5 mismatches, >2 indels in the aligned read, >4 bp indel size. Alignments were sorted, indexed and read groups were added using Picard tools version 1.130 (Wysoker *et al.*, 2013). After *bam* file cleaning, 72.2 % of the aligned reads were kept (Table S1). Local realignment near indels was performed using the Genome Analysis Toolkit (GATK version 3.3-0-g37228af; McKenna *et al.*, 2010; Huang *et al.*, 2012)⁠. Working with alignment files and thus genotype likelihoods rather than called genotypes allows to get rid of genotype calls uncertainty and is therefore a statistical advantage for downstream analyses (Nielsen *et al.*, 2012; Korneliussen *et al.*, 2014)⁠. When possible, we accordingly performed subsequent analyses based on alignment files. However, some analyses require genotype calls as input and we hence called genotypes and filter variants based on different criteria.

We called genotypes using GATK with the algorithm *UnifiedGenotyper*. This led to 1,157,389 variants among the 68 *Olea europaea* accessions. The following criteria were then employed to subsequently filter sites: sites with quality < 30, depth < 20, quality by depth QD < 1.5, high Fisher strand bias FS > 60. Sites close to indels (5 bp) and in cluster of more than three in window size of 10 bp were also filtered out. Genotypes with a genotype quality below 20 were set as missing. Finally, sites where < 50% of the individuals were scored were removed. This led to 583,455 SNPs displaying an average of 12.94% of missing genotypes (Table S1) among the 68 *Olea europaea* accessions as 49.59% SNPs where excluded during the filtering phase. When the two accessions of *Olea europaea* subsp. *cuspidata* were excluded prior to filtering, this led to a total of 536,341 SNPs, covering 25,836 contigs over the 45,389 contigs (57%), with an average of 20.76 SNPs per contig.

**Methods S3** **Inference of relatedness among accessions.**

To gain knowledge into the relationships among olive accessions and confirm or identify clones, we inferred their degree of genetic relatedness. Using NGSrelate (Korneliussen & Moltke, 2015) we calculated maximum-likelihood estimates of k_0_, k_1_ and k_2_ which are the probabilities that two individuals share respectively 0, 1 or 2 alleles identical by descent⁠. The relatedness *r* was then calculated and a heatmap of relatedness was generated with the *R* package *igraph* (Csardi & Nepusz, 2006) (Fig. S2. )⁠. About 60% of the pairs of accessions display no relatedness (*r* < 0.05) and only 1 % displays a relatedness above 0.50. The three known pairs of clones (OGMed_025/OGMed_026, OGMed_018/ OGMed_055 and OGMed_029/ OGMed_046) display relatedness above 92 % and we thus used this threshold to identify putative clones. Surprisingly, OGMed_052 is a clone of OGMed_025 and OGMed_026 (var. Kato Dris and Menikon both from Cyprus, respectively; *r* = 1 in both cases). No other redundant genotypes were identified. For the subsequent analyses, we therefore removed OGMed_026, OGMed_052, OGMed_055 and OGMed_029 in order to keep only unique genotypes in further analyses.

**Methods S4 Inference of Site Frequency Spectra and calculation of diversity statistics.**

We generated individual or population Site Frequency Spectra (SFS) with ANGSD (Korneliussen *et al.*, 2014) using the genotype likelihood as implemented in GATK (-*GL* 2 option) and with the following filtering options: minimum mapping quality above 30 (-*minMapQ* option) and minimum base quality above 20 (-*minQ* option). Then, the site allele frequency likelihoods were optimized to generate an estimate of the SFS following the method described in Nielsen *et al.* (2012). This approach allows an estimation of diversity statistics based on genotype likelihoods which is more accurate than using genotype calls, especially in low coverage regions (Nielsen *et al.*, 2012; Korneliussen *et al.*, 2014). We determined the observed heterozygosity with individual folded SFSs by dividing the second bin of each SFS (= the number of sites with one alternative allele) by the total number of sites (= the sum of the SFS). For each population, we averaged over all accessions the individual observed heterozygosity to get an observed heterozygosity for each population. The expected heterozygosity was calculated based on population unfolded SFSs using ngsStat utility from the ngsPopGen software (Fumagalli, 2013), while we generated estimates of thetas and Tajima’s *D* from population folded SFSs using thetaStat utility implemented in ANGSD.

**Methods S5 Annotation of the transcriptome reference sites as synonymous or non-synonymous.**

We annotated sites from the transcriptome reference (Sarah *et al.*, 2017) as synonymous (i.e., not causing a change in the amino acid) and non-synonymous (when the amino acid is altered). We first extracted putative coding sequence (CDS) and coding frame from the transcript sequences employing FrameDP version 1.2.2 (Gouzy et al., 2009) with default parameters. We processed both the *Universal Protein Resource* (UniProt, The UniProt Consortium, 2014) and the Arabidopsis *Information Resource* (TAIR, Berardini *et al.*, 2015) as reference databases for the underlying Hidden Markov Model (HMM). Over the 45,389 contigs of the transcriptome reference, 30,697 were found to contain a CDS and we further filtered out contigs for which FrameDP found several CDS. Sites were categorized as first, second, or third positions of codons and based on the genetic code, their level of degeneracy (zero-fold, 2-fold, or 4-fold degenerate) was deducted using a custom Perl script. We treated as synonymous all 4-fold degenerate sites (1,891,269 sites), while 0-fold degenerate sites (7,689,634 sites) were treated as non-synonymous.

**Supplementary References**

**Bandelt HJ, Forster P, Rohl A. 1999**. Median-joining networks for inferring intraspecific phylogenies. *Molecular Biology and Evolution* **16**: 37–48.

**Berardini TZ, Reiser L, Li D, Mezheritsky Y, Muller R, Strait E, Huala E. 2015**. The arabidopsis information resource: Making and mining the “gold standard” annotated reference plant genome. *Genesis* **53**: 474–485.

**Besnard G, El Bakkali A, Haouane H, Baali-Cherif D, Moukhli A, Khadari B. 2013a**. Population genetics of Mediterranean and Saharan olives: geographic patterns of differentiation and evidence for early generations of admixture. *Annals of Botany* **112**: 1293–1302.

**Besnard G, Hernández P, Khadari B, Dorado G, Savolainen V, Hernandez P, Khadari B, Dorado G, Savolainen V. 2011**. Genomic profiling of plastid DNA variation in the Mediterranean olive tree. *BMC Plant Biology* **11**: 80.

**Besnard G, Khadari B, Navascués M, Fernández-Mazuecos M, El Bakkali A, Arrigo N, Baali-Cherif D, Brunini-Bronzini de Caraffa V, Santoni S, Vargas P, et al. 2013b**. The complex history of the olive tree: from Late Quaternary diversification of Mediterranean lineages to primary domestication in the northern Levant. *Proceedings of the Royal Society, Series B* **280**: 20122833.

**Clément Y, Sarah G, Holtz Y, Homa F, Pointet S, Contreras S, Nabholz B, Sabot F, Sauné L, Ardisson M, et al. 2017**. Evolutionary forces affecting synonymous variations in plant genomes. *PLOS Genet.* **13**, e1006799.

**Csardi G, Nepusz T. 2006**. The igraph software package for complex network research. *InterJournal Complex System*: **1695**.

**Fumagalli M. 2013**. Assessing the effect of sequencing depth and sample size in population genetics inferences. *PloS ONE* **8**: e79667.

**Gouzy J, Carrere S, Schiex T. 2009**. FrameDP: sensitive peptide detection on noisy matured sequences. Bioinformatics **25**: 670–671.

**Haouane H, El Bakkali A, Moukhli A, Tollon C, Santoni S, Oukabli A, El Modafar C, Khadari B. 2011.** Genetic structure and core collection of the World Olive Germplasm Bank of Marrakech: towards the optimised management and use of Mediterranean olive genetic resources. *Genetica* **139**, 1083–1094.

**Huang X, Kurata N, Wei X, Wang Z-X, Wang A, Zhao Q, Zhao Y, Liu K, Lu H, Li W, et al. 2012**. A map of rice genome variation reveals the origin of cultivated rice. Nature **490**: 497–501.

**Korneliussen TS, Albrechtsen A, Nielsen R. 2014**. ANGSD: Analysis of Next Generation Sequencing Data. *BMC Bioinformatics* **15**: 356.

**Korneliussen TS, Moltke I. 2015**. NgsRelate: a software tool for estimating pairwise relatedness from next-generation sequencing data. Bioinformatics **31**: 4009–11.

**Li H, Durbin R. 2010**. Fast and accurate long-read alignment with Burrows-Wheeler transform. *Bioinformatics* **26**: 589–595.

**Li H, Handsaker B, Wysoker A, Fennell T, Ruan J, Homer N, Marth G, Abecasis GR, Durbin R, 1000 Genome Project Data Processing Subgroup. 2009**. The Sequence Alignment/Map format and SAMtools. *Bioinformatics* **25**: 2078–2079.

**Martin M. 2011**. Cutadapt removes adapter sequences from high-throughput sequencing reads. *EMBnet.journal* **17**: 10.

**McKenna A, Hanna M, Banks E, Sivachenko 1Andrey, Cibulskis K, Kernytsky A, Garimella K, Altshuler D, Gabriel S, Daly MJ, et al. 2010**. The Genome Analysis Toolkit: a MapReduce framework for analyzing next-generation DNA sequencing data. *Genome Research* **20**: 1297–1303.

**Nielsen R, Korneliussen T, Albrechtsen A, Li Y, Wang J. 2012**. SNP calling, genotype calling, and sample allele frequency estimation from New-Generation Sequencing data. (P Awadalla, Ed.). *PLoS ONE* **7**: e37558.

**Parra G, Bradnam K, Korf I. 2007**. CEGMA: a pipeline to accurately annotate core genes in eukaryotic genomes. *Bioinformatics* **23**: 1061–1067.

**Piruat JI, Aguilera A. 1998**. A novel yeast gene, THO2, is involved in RNA pol II transcription and provides new evidence for transcriptional elongation-associated recombination. *EMBO Journal* **17**: 4859–4872.

**Sarah G, Homa F, Pointet S, Contreras S, Sabot F, Nabholz B, Santoni S, Sauné L, Ardisson M, Chantret N, et al. 2017**. A large set of 26 new reference transcriptomes dedicated to comparative population genomics in crops and wild relatives. *Molecular Ecology Resources* **17**: 565–580.

**The UniProt Consortium. 2014**. UniProt: a hub for protein information. *Nucleic Acids Research* **43**: D204-212.

**Vanholme R, Cesarino I, Rataj K, Xiao Y, Sundin L, Goeminne G, Kim H, Cross J, Morreel K, Araujo P, et al. 2013**. Caffeoyl Shikimate Esterase (CSE) Is an Enzyme in the Lignin Biosynthetic Pathway in Arabidopsis. *Science* **341**: 1103–1106.

**Vogelstein B, Lane D, Levine AJ. 2000**. Surfing the p53 network. *Nature* **408**: 307–310.

**Wikstrom M, Krab K, Saraste M. 1981**. Cytochrome Oxidase—A Synthesis. Ac*ademic Press (February 1, 1982),* New York.

**Wysoker A, Tibbetts K, Fennell T. 2013**. Picard tools version 1.90. http://picard.sourceforge.net.
