# Supplementary material for: Evolutionary transcriptomics reveals the origins of olives and the genomic changes associated with their domestication
Source: Plant J. 2019 Jul 11;100(1):143–57. doi: 10.1111/tpj.14435 (PMC6851578; doi:10.1111/tpj.14435)
Supplement: Supplementary file 6 — Appendix S1. Detailed structure and diversity of oleaster populations. [file TPJ-100-143-s006.docx]

**Appendix S1 Detailed structure and diversity of oleaster populations**

Most of our sampling (22 over 27 accessions) for oleasters originate from the region of the presumed domestication center in Syria/Turkey (Table S1). Five accessions were sampled in various locations in Syria and Izmir, Turkey before being transplanted in a germplasm collection, in Porquerolles Island, France, where we collected the tissue for the RNA-sequencing. Seventeen other accessions were sampled *in situ*, in three locations in Turkey. The remaining five eastern accessions belong to the western populations.

Eastern and western populations show a high level of differentiation (*Fst*=0.167 ± 0.313, Table 2), while, within these two populations, structuration is limited (Fig. S3). We estimated the diversity within the five distinct eastern oleaster populations by calculating the observed heterozygosity (*Ho*), as explained in Methods S4. Accessions originally from Syria (see Table S1) show an average *Ho* of 0.00397, those from Bogazkirim and Izmir, Turkey of 0.00395, those from Kavakli of 0.00402 and those from Korucak of 0.00400. Nevertheless, these differences are not significant (Tukey test, *P* > 0.05).
